# Supplementary material for: HDX-MS reveals dysregulated checkpoints that compromise discrimination against self RNA during RIG-I mediated autoimmunity
Source: Nat Commun. 2018 Dec 18;9:5366. doi: 10.1038/s41467-018-07780-z (PMC6299088; doi:10.1038/s41467-018-07780-z)
Supplement: Supplementary file 1 — Supplementary Information [file 41467_2018_7780_MOESM1_ESM.pdf]

Supplementary Information.

HDX-MS reveals dysregulated checkpoints that compromise discrimination against self RNA during RIG-I mediated autoimmunity

Jie Zheng[1#*], Chen Wang[2#], Mi Ra Chang[1], Swapnil C. Devarkar[3], Brandon Schweibenz[3], Gogce C. Crynen[4], Ruben D. Garcia-Ordonez[1], Bruce D. Pascal[1,5], Scott J. Novick[1], Smita S. Patel[3], Joseph Marcotrigiano[2*], Patrick R. Griffin[1*]

[1]The Scripps Research Institute, Department of Molecular Medicine, Jupiter, FL 33458, USA

[2]Structural Virology Section, National Institute of Allergy and Infectious Diseases, National Institutes of Health, Bethesda, MD 20892, USA

[3]Robert Wood Johnson Medical School, Rutgers University, Piscataway, NJ 08854, USA

[4]The Center for Computational Biology, The Scripps Research Institute, Jupiter, FL 33458, USA

[5]Omics Informatics LLC, Honolulu, HI96813, USA

*To whom correspondence could be addressed:

pgriffin@scripps.edu

joseph.marcotrigiano@nih.gov

jzheng@scripps.edu

[#]denotes equal contribution.

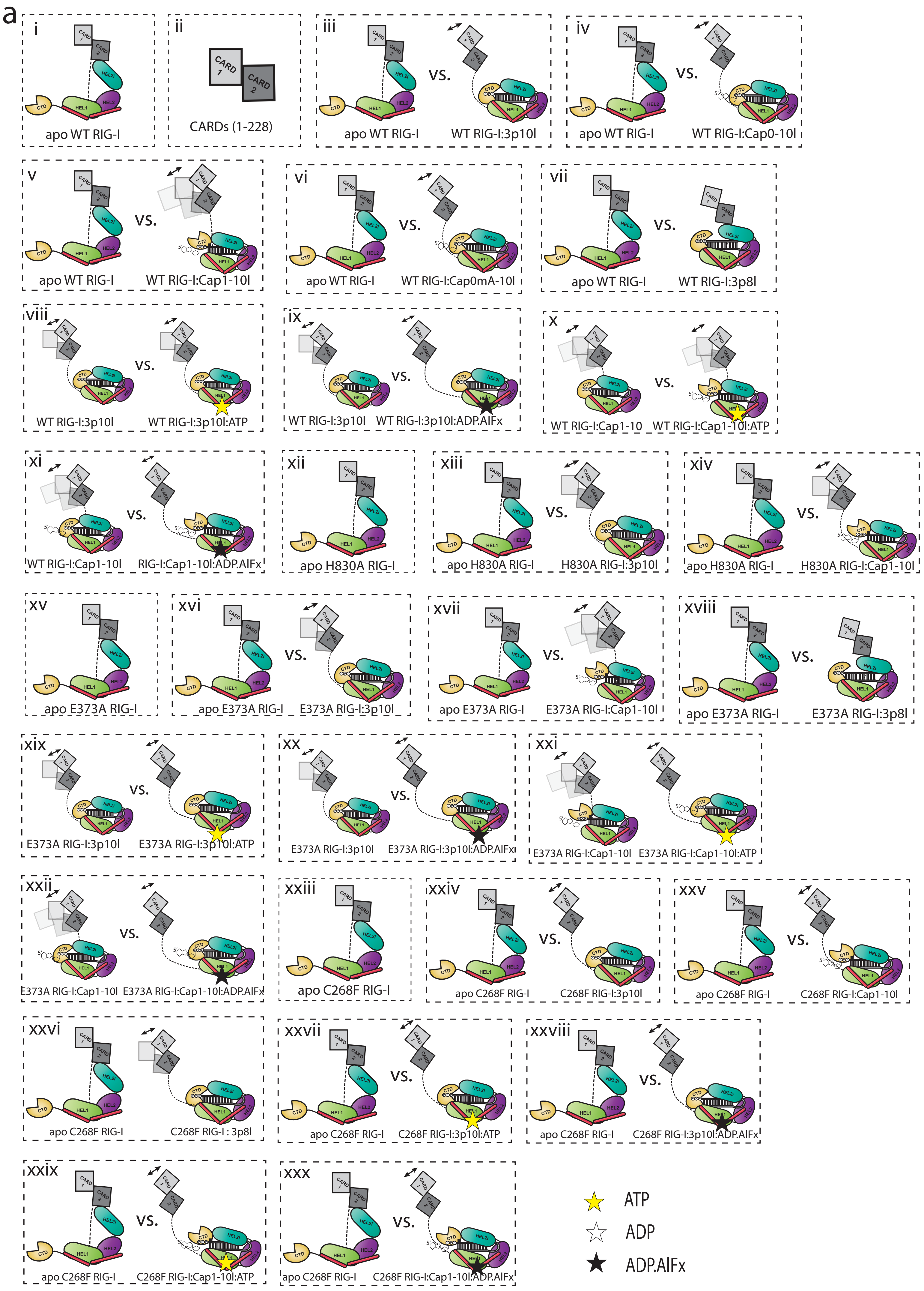

Supplementary Fig. 1a

Supplementary Fig. 1b

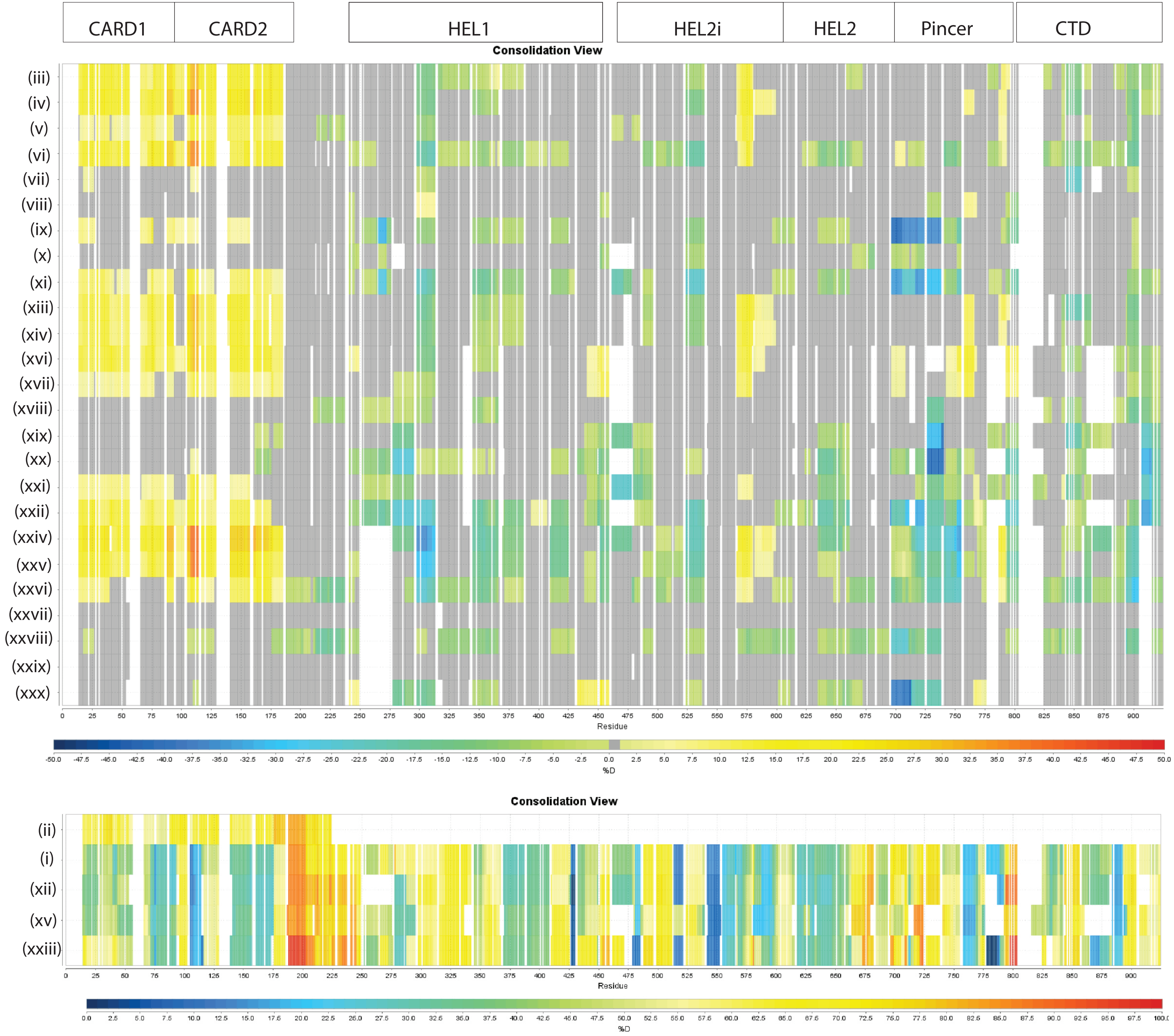

Supplementary Fig. 1c

WT RIG-I peptide sequence	charge	start	end	structure	(i)	(ii)	(iii)	(iv)	(v)	(vi)	(vii)	(viii)	(ix)	(x)	(xi)
QDYIRKTLDPTYIL	3	13	26		36 (3)	65 (3)	15 (5)	21 (4)	9 (4)	17 (3)	4 (4)*	-1 (4)*	9 (5)	-1 (2)*	10 (4)
IRKTLDPTYIL	3	16	26		32 (2)	63 (5)	21 (2)	23 (5)	10 (5)	22 (3)	6 (4)	-1 (4)*	7 (3)	0 (2)*	13 (4)
SYMAPWFRE	2	27	35		41 (3)	72 (5)	21 (4)	24 (4)	11 (3)	22 (3)	2 (4)*	-4 (4)*	3 (3)*	0 (2)*	15 (3)
YMAPWFREEEVQ	2	28	39		44 (2)	76 (4)	19 (4)	23 (3)	10 (2)	19 (3)	3 (3)*	-2 (5)*	2 (4)*	-1 (2)*	9 (3)
VQYIQA	2	38	43		25 (2)	56 (4)	20 (4)	18 (4)	3 (3)*	13 (4)	0 (2)*	-5 (4)	1 (5)*	2 (3)*	7 (5)
YIQAEKNNKGPMEA	3	40	53		43 (4)	71 (5)	13 (5)	17 (3)	7 (4)	12 (7)	-2 (4)*	-2 (3)*	1 (4)*	-2 (4)*	1 (6)*
EKNNKGPMEAATL	2	44	56		39 (3)	55 (7)	15 (4)	15 (4)	5 (4)	16 (5)	2 (5)*	-3 (3)*	3 (5)*	-1 (3)*	8 (5)
LQEEGWFRGFL	2	64	74	CARD1	47 (4)	56 (8)	11 (5)	17 (4)	8 (4)	9 (4)	3 (4)*	2 (5)*	9 (4)	-3 (2)*	0 (4)*
QEEGWFRGFL	2	65	74		46 (2)	53 (4)	10 (3)	9 (6)	10 (5)	6 (6)	4 (7)*	-2 (4)*	6 (3)	-1 (3)*	4 (4)*
FRGFLDA	2	70	76		15 (1)		7 (2)	18 (2)	11 (3)	24 (3)	1 (3)*	4 (3)*	14 (5)	1 (2)*	12 (3)
DALDHAGYSGL	2	75	85		27 (2)	54 (5)	16 (3)	20 (3)	9 (3)	21 (3)	2 (4)*	-1 (4)*	5 (3)	0 (2)*	7 (5)
LDHAGYSGL	2	77	85		32 (2)	50 (6)	11 (4)	11 (4)	5 (3)*	14 (4)	4 (5)*	-1 (5)*	5 (4)*	-1 (3)*	7 (3)
YEAIESW	1	86	92		22 (2)	66 (4)	29 (3)	32 (3)	19 (4)	30 (3)	2 (4)*	-2 (4)*	7 (3)	-1 (3)*	16 (4)
YEAIESWD	1	86	93		19 (2)	64 (6)	25 (4)	32 (3)	16 (3)	33 (3)	2 (4)*	1 (5)*	10 (6)	0 (3)*	17 (3)
FKKIEKLEE	3	94	102		64 (3)	72 (5)	8 (4)	13 (3)	4 (2)*	6 (3)	-4 (5)*	-2 (4)*	3 (4)*	-2 (2)*	4 (4)*
YRLLL	2	103	107		8 (1)	52 (3)	21 (2)	26 (2)	7 (2)	28 (2)	3 (5)*	0 (4)*	7 (7)	1 (3)*	20 (2)
YRLLLKRLQPEF	3	103	114		13 (1)	60 (4)	31 (3)	38 (4)	18 (5)	34 (3)	5 (4)*	2 (6)*	6 (3)	0 (3)*	13 (5)
LLKRLQPEF	2	106	114	Latch region	17 (3)	66 (6)	30 (5)	37 (5)	21 (4)	37 (5)	6 (4)	-1 (5)*	7 (4)	0 (3)*	15 (5)
LLKRLQPEF	3	106	114		14 (3)	65 (6)	35 (3)	38 (6)	17 (7)	38 (4)	6 (4)	-3 (4)*	7 (3)	1 (4)*	19 (9)
KRLQPEF	2	108	114		21 (3)	76 (5)	31 (5)	34 (5)	18 (6)	34 (5)	6 (5)	-2 (6)*	4 (5)*	1 (4)*	13 (7)
KTRIIPTDIISDL	2	115	127		53 (3)	73 (5)	19 (4)	22 (3)	13 (3)	19 (3)	2 (4)*	-3 (4)*	3 (4)*	-2 (2)*	6 (3)
KTRIIPTDIISDL	3	115	127		46 (2)	72 (5)	19 (4)	21 (3)	12 (3)	18 (3)	3 (4)*	-3 (4)*	3 (4)*	-2 (3)*	6 (4)
KTRIIPTDIISD	3	115	126		47 (2)	71 (4)	15 (5)	12 (4)	8 (4)	15 (4)	1 (4)*	2 (5)*	2 (5)*	-2 (3)*	7 (4)
ILQICSTKGMMAGAEKLVE	3	139	157		28 (1)	57 (4)	22 (2)	25 (2)	12 (2)	23 (2)	5 (3)	-1 (4)*	7 (2)	0 (2)*	13 (2)
LLRSDKENWPKTLKL	4	159	173	CARD2	36 (0)	75 (3)	12 (7)	29 (2)	14 (4)	12 (3)	-2 (3)*	-1 (5)*	-1 (2)*	0 (2)*	13 (5)
RSDKENWPKTLKL	3	161	173		34 (4)	65 (5)	16 (6)	18 (4)	10 (5)	20 (5)	3 (0)*	0 (4)*	4 (4)*	-2 (16)*	10 (5)
RSDKENWPKTLKLAL	4	161	175		28 (3)	59 (5)	23 (4)	26 (3)	10 (5)	21 (4)	-3 (4)*	3 (4)*	2 (4)*	3 (3)*	
ALEKERNKFSEL	3	174	185		57 (1)	82 (6)	19 (3)	20 (3)	10 (3)	17 (3)	0 (4)*	-3 (4)*	4 (5)*	-3 (5)*	6 (5)
EKERNKFSEL	3	176	185		56 (2)	77 (5)	13 (4)	15 (4)	7 (3)	11 (4)	0 (3)*	-2 (4)*	4 (5)*	-2 (3)*	5 (4)
WIVEKGIKDVETEDLED	2	186	202		88 (2)	87 (6)	5 (2)	0 (4)*	-2 (5)*	-1 (5)*	-2 (3)*	-1 (2)*	-3 (2)*	0 (1)*	-2 (5)*
WIVEKGIKDVE	3	186	196		86 (4)	85 (6)	2 (3)*	2 (4)*	0 (4)*	-1 (7)*	-1 (3)*	-1 (3)*	0 (1)*	-2 (4)*	-2 (5)*
WIVEKGIKDVETEDL	3	186	200		86 (1)	84 (3)	4 (6)*	2 (6)*	-1 (6)*	-1 (6)*	-1 (6)*	-4 (5)*	-2 (6)*	-3 (5)*	-1 (5)*
IVEKGIKDVETEDL	3	187	200		84 (1)	81 (6)	5 (2)*	3 (4)*	2 (4)*	-1 (4)*	-1 (6)*	-4 (3)*	5 (2)	-2 (4)*	0 (3)*
EDKMETSDIQIF	2	201	212	linker	70 (4)	75 (6)	4 (5)*	3 (4)*	2 (5)*	1 (5)*	-2 (5)*	-2 (4)*	0 (6)*	-2 (5)*	1 (4)*
FYQEDPECQNL	2	212	222		73 (1)	63 (5)	3 (3)*	1 (3)*	-10 (5)	-4 (4)*	-1 (3)*	-1 (3)*	5 (5)*	-2 (5)*	1 (5)*
YQEDPECQNL	2	213	222		67 (3)	70 (5)	2 (5)*	-2 (5)*	-11 (6)	1 (5)*	1 (2)*	-3 (5)*	-1 (5)*	-2 (5)*	-3 (3)*
SENSCPPSEVSDTNL	2	223	237		71 (5)		5 (6)*	-2 (4)*	-9 (7)	-2 (5)*	-2 (6)*	-4 (5)*	-3 (5)*	-2 (6)*	-1 (5)*
VSDTNL	1	232	237		72 (5)		4 (6)*	-2 (3)*	-13 (7)	-2 (6)*		-3 (5)*	-2 (4)*	-2 (6)*	-1 (5)*
YSPFKPRNYQLE	2	238	249		79 (4)		0 (4)*	3 (4)*	-1 (5)*	-6 (4)	1 (5)*	-4 (5)*	-3 (4)*	-6 (4)	-3 (4)*
YSPFKPRNYQLE	3	238	249	HEL1	62 (3)		1 (4)*	3 (4)*	-2 (4)*	-6 (4)	2 (5)*	-4 (5)*	-4 (4)*	-6 (3)	-4 (4)*
YSPFKPRN	3	238	245		62 (3)		4 (6)*	-1 (3)*	-2 (5)*	3 (6)*	-3 (2)*	-9 (6)	-10 (5)	-7 (5)	-11 (4)
LALPAMKGKNTIICAPTGCGKTFVSLL	3	250	276		46 (2)		0 (3)*	3 (2)*	4 (3)*	-4 (3)*	3 (3)*	-1 (4)*	-12 (3)	3 (7)*	-9 (4)
LALPAMKGKNTIICAPTGCGKTFVSLL	4	250	276		51 (1)		-5 (1)*	3 (1)*	-3 (3)*	2 (6)*	0 (5)*	-3 (4)*	-11 (3)	-4 (3)*	
APTGCGKTF	2	264	272		72 (3)		2 (4)*	0 (3)*	-3 (8)*	4 (5)*	-5 (3)*		-46 (6)	-10 (5)	-32 (5)
ICEHHLKKFPQGQKGKVVF	3	277	295		34 (2)		2 (3)*	1 (2)*	-2 (3)*	-1 (2)*	-3 (3)*	-1 (3)*	-3 (2)*		-3 (3)*
PQGQKGKVVF	3	286	295		63 (3)		-4 (4)*	-3 (3)*	4 (5)*	4 (3)*	-2 (2)*	0 (4)*	2 (4)*	-2 (4)*	0 (6)*
FANQIPVYEQQ	2	296	306		57 (2)		-12 (3)	-12 (4)	-10 (6)	-16 (4)	-4 (4)*	6 (5)	-9 (5)	-4 (5)*	-26 (6)
FANQIPVYEQQKSVF	2	296	310	motif Ia	56 (1)		-14 (3)	-12 (5)	-6 (4)	-18 (3)	-6 (3)	6 (5)	-10 (5)	-3 (4)*	-22 (6)
FANQIPVYEQQKSVFSKY	3	296	313		53 (1)		-13 (3)	-12 (5)	-14 (4)	-21 (4)	-6 (4)	6 (3)	-10 (5)	4 (5)*	-19 (6)
FERHGYRVTGISGATAENVPVEQIVENND	3	314	342		59 (2)		-14 (2)	-3 (3)*	-2 (4)*	-6 (3)	-1 (4)*	-2 (4)*	0 (3)*		-1 (3)*
GYRVTGISGATAENVPVEQIVENN	4	318	341		69 (2)		-3 (2)*	0 (2)*	0 (6)*	-6 (3)	-3 (5)*	3 (2)*	1 (4)*	-1 (3)*	4 (0)*
YRVTGISGATAENVPVEQIVE	3	319	339		72 (4)		3 (5)*	0 (6)*	-3 (4)*	-3 (6)*	-3 (3)*	-3 (4)*	-4 (4)*	-5 (5)	-1 (7)*
IIILTPQILVNNLKKGTIPSL	2	343	363		28 (2)		-6 (4)	-8 (3)	-3 (4)*	-10 (3)	-3 (3)*	-2 (4)*	-10 (3)	-4 (4)*	-16 (4)
IIILTPQILVN	2	343	353		41 (2)		-3 (3)*	-6 (4)	-3 (2)*	-9 (4)	3 (3)*	1 (3)*	-7 (2)	-4 (2)*	-13 (2)
IIILTPQILVNNL	2	343	355		58 (2)		-8 (3)	-11 (3)	-5 (3)	-11 (3)	-1 (3)*	-1 (3)*	-10 (2)	-5 (3)	-20 (3)
IIILTPQILVNNLKKGTIPSL	3	343	363	motif Ic	58 (2)		-6 (3)	-9 (3)	-3 (4)*	-11 (3)	-3 (3)*	-2 (4)*	-9 (3)	-5 (4)	-16 (3)
IIILTPQILVNNLKKGTIPSLSI	3	343	365		57 (2)		-6 (3)	-7 (2)	-2 (3)*	-9 (3)	-3 (3)*	-2 (4)*	-9 (3)	-5 (4)	-15 (3)
IIILTPQILVNNLKKGTIPSLSIF	3	343	366		54 (2)		-5 (3)	-7 (2)	-1 (2)*	-8 (2)	-1 (3)*	-1 (4)*	-8 (3)	-5 (4)*	-14 (2)
LMIFDECHNTSKQHPYNM	3	368	385		29 (2)		-6 (2)	-4 (2)*	-2 (2)*	-10 (2)	3 (5)*	2 (3)*	-6 (3)	-1 (2)*	-10 (3)
IFDECHNTSKQHPYNMIM	2	370	387	motif IIa	30 (1)		-5 (2)	-4 (2)*	-1 (2)*	-10 (2)	2 (5)*	3 (7)*	-7 (2)	2 (7)*	-10 (3)
IFDECHNTSKQHPYNMIM	3	370	387		29 (2)		-6 (2)	-4 (2)*	-2 (2)*	-10 (2)	3 (5)*	2 (3)*	-6 (3)	-1 (2)*	-10 (3)
FNYLDQKLGGSSGPLPQVIGL	2	388	408		33 (2)		1 (2)*	4 (3)*	2 (4)*	-5 (2)	3 (3)*	0 (4)*	3 (3)*	0 (3)*	3 (4)*
FNYLDQKLGGSSGPLPQVIG	2	388	407		33 (1)		2 (2)*	3 (2)*	2 (4)*	-6 (4)	4 (4)*	-1 (4)*	2 (2)*	-1 (3)*	3 (4)*
DQKLGGSSGPLPQVIGL	2	392	408		39 (2)		0 (2)*	4 (3)*	1 (4)*	-3 (2)*	1 (3)*	0 (3)*	2 (3)*	-1 (3)*	2 (4)*
TASVGVGDAKNTDEALD	2	409	425		61 (3)		-2 (4)*	-3 (4)*	-1 (4)*	-7 (4)	-3 (5)*	-1 (5)*	-8 (4)	-4 (4)*	-11 (4)
TASVGVGDAKNTDEAL	2	409	424		59 (3)		-3 (4)*	-3 (4)*	-2 (5)*	-6 (4)	-4 (2)*	-1 (5)*	-8 (4)	-5 (4)*	-10 (4)
DYICKL	1	425	430		1 (1)		-1 (1)*	1 (1)*	1 (1)*	-3 (1)*	0 (2)*	0 (0)*	0 (2)*	0 (1)*	0 (0)*
DYICKL	2	425	430		3 (1)		-1 (1)*	-1 (1)*	-3 (1)*	-2 (1)*	0 (1)*	1 (1)*	1 (2)*	0 (1)*	0 (1)*
CASLDASVIATVKHNLEEL	2	431	449		43 (2)		-5 (3)*	-1 (3)*	0 (3)*	-5 (2)*	3 (4)*	1 (4)*	2 (3)*	-3 (2)*	-2 (3)*
SVIATVKHNLEE	2	437	448		55 (3)		-2 (3)*	-1 (3)*	1 (3)*	-2 (3)*	-3 (4)*	-3 (3)*	-2 (3)*	-4 (3)*	1 (2)*
EQVVYKPQKF	3	450	459		55 (4)		3 (4)*	3 (4)*	2 (5)*	5 (5)*	4 (6)*	-8 (3)	-5 (3)*	-12 (4)	2 (6)*
VVYKPQKF	2	452	459		60 (3)		1 (4)*	4 (4)*	4 (4)*	2 (4)*	-1 (6)*	-3 (4)*	-3 (4)*	-6 (4)	-4 (4)*
VVYKPQKF	3	452	459		61 (2)		2 (4)*	0 (4)*	1 (4)*	1 (4)*	0 (6)*	-4 (6)*	-1 (3)*	-6 (4)	-5 (4)
FRKVESRISDKFKYIIAQL	3	460	478		39 (3)		1 (3)*	-1 (3)*	-5 (4)	-2 (3)*	-1 (6)*	-1 (3)*	-4 (2)*		-13 (6)
MRDTESL	2	479	485		5 (2)		-1 (2)*	-1 (2)*	-3 (2)*	-2 (2)*	-1 (2)*	0 (1)*	0 (4)*	0 (1)*	0 (1)*
AKRICKDLENL	3	486	496	HEL2i	69 (3)		-4 (5)*	-4 (4)*	-1 (4)*	-11 (4)	2 (6)*	-2 (5)*	-1 (5)*	-4 (3)*	-6 (4)
SQIQNREFGTQKYEQW	3	497	512		68 (2)		-1 (4)*	-1 (4)*	-1 (4)*	-7 (4)	0 (5)*	-2 (4)*	1 (3)*	-2 (4)*	0 (4)*
SQIQNREFGTQKYEQ	3	497	511		62 (3)		-1 (4)*	-4 (3)*	-1 (2)*	-13 (3)	-2 (5)*	-3 (4)*	-1 (3)*	-2 (4)*	-1 (4)*
WIVTVQKACM	2	512	521		9 (2)		-3 (2)*	-2 (3)*	0 (2)*	-8 (2)	5 (2)*	-3 (5)*	0 (5)*	1 (7)*	-2 (3)*
IVTVQKACM	2	513	521		13 (2)		-4 (2)*	-3 (3)*	-2 (3)*	-9 (2)	5 (3)*	2 (4)*	3 (5)*	-1 (2)*	1 (3)*
VFQMPDKDEESRICKA	3	522	537		60 (2)		-9 (3)	-12 (3)	-4 (4)*	-15 (3)	1 (6)*	-2 (4)*	-11 (3)	-8 (3)	-24 (3)
VFQMPDKDEESRICKALF	4	522	539	CTD:HEL2i	51 (2)		-6 (3)	-10 (2)	-1 (2)*	-14 (3)	3 (5)*	-2 (4)*	-9 (3)	-7 (3)	-21 (2)
FQMPDKDEESRICKA	3	523	537		62 (3)		-7 (4)	-18 (3)	-4 (4)*	-19 (4)	2 (8)*	-2 (4)*	-11 (3)	-8 (3)	-24 (3)
FQMPDKDEESRICKALF	4	523	539		49 (2)		-6 (3)	-11 (3)	-3 (3)*	-20 (4)	2 (4)*	-2 (4)*	-10 (3)	-7 (2)	-24 (2)
LYTSHLRKYNDAL	3	540	552		11 (2)		-1 (3)*	5 (5)*	2 (6)*	-5 (3)	3 (2)*	1 (5)*	2 (2)*	0 (4)*	0 (6)*
LYTSHLRKYNDAL	4	540	552		12 (2)		-1 (4)*	3 (5)*	2 (5)*	-5 (3)*	4 (3)*	2 (4)*	2 (3)*	1 (4)*	-1 (5)*
YTSHLRKYNDAL	2	541	552		13 (2)		-1 (3)*	5 (5)*	1 (6)*	-1 (4)*	4 (2)*	2 (5)*	3 (4)*	0 (5)*	2 (7)*
IISEHARMKDALD	3	553	565		24 (2)		-2 (2)*	1 (3)*	-1 (3)*	-5 (3)	3 (4)*	1 (3)*	2 (3)*	0 (2)*	-1 (3)*
HARMKDALD	2	557	565		30 (2)		0 (3)*	0 (3)*	-2 (3)*	-4 (2)*	3 (3)*	0 (3)*	0 (3)*	1 (3)*	0 (2)*
HARMKDALD	3	557	565		32 (2)		-1 (3)*	-1 (3)*	-2 (3)*	-5 (3)*	0 (3)*	0 (3)*	0 (3)*	-2 (2)*	-1 (2)*
DYLKDFFSNVRAAGF	3	565	579		34 (2)		14 (3)	12 (4)	9 (3)		2 (3)*	0 (5)*	2 (2)*	-1 (4)*	2 (3)*
YLKDFFSNV	2	566	574		16 (1)		10 (2)	14 (3)	10 (2)	9 (2)	5 (4)*	0 (2)*	2 (2)*	-1 (1)*	1 (2)*
YLKDFFSNVRAAGF	3	566	579	Gate motif	35 (2)		14 (4)	17 (3)	12 (3)	11 (3)	3 (4)*	-3 (3)*	1 (3)*	-4 (2)*	-1 (2)*
FFSNV	1	570	574					30 (2)	21 (3)	6 (3)	3 (3)*	0 (5)*	2 (3)*	-2 (2)*	3 (3)*
FFSNVRAAGFD	2	570	580		49 (1)		15 (5)	18 (4)	15 (5)	14 (4)	-1 (4)*	3 (6)*	2 (3)*		-2 (8)*
FFSNVRAAGF	2	570	579		49 (2)		17 (3)	19 (3)	13 (3)	17 (3)	4 (4)*	-2 (4)*	0 (4)*	-1 (4)*	1 (5)*

WT RIG-I peptide sequence	charge	start	end	structure	(i)	(ii)	(iii)	(iv)	(v)	(vi)	(vii)	(viii)	(ix)	(x)	(xi)
DEIEQDLTQRFEEKLQE	3	580	596	HEL2i	25 (2)		3 (2)*	11 (3)	2 (3)*	2 (2)*	5 (3)*	0 (2)*	0 (2)*	-3 (2)*	-1 (2)*
DEIEQDLTQRFEEKLQEL	3	580	597		25 (1)		4 (2)*	8 (1)	3 (2)*	2 (3)*	3 (2)*	0 (3)*	0 (2)*	-1 (1)*	0 (2)*
DEIEQDLTQRFEEKLQELES	3	580	599		27 (1)		4 (2)*	7 (1)	3 (2)*	1 (2)*	3 (3)*	0 (3)*	0 (3)*	-1 (2)*	-2 (2)*
QELESVSRDPSNENPKLED	3	595	613		47 (2)		2 (3)*	1 (2)*	1 (5)*	-3 (3)*	-1 (3)*	-1 (4)*	-7 (4)	-3 (4)*	-6 (5)
ESVSRDPSNENPKLEDLC	3	598	615		40 (1)		0 (1)*	3 (3)*	2 (3)*	0 (2)*	4 (6)*	-3 (3)*	-6 (7)	1 (3)*	-6 (3)
VSRDPSNENPKLED	2	600	613		58 (2)		3 (4)*	2 (4)*	1 (4)*	-1 (5)*	-2 (5)*	-3 (4)*	-7 (4)	-2 (4)*	-6 (4)
VSRDPSNENPKL	3	600	611		51 (3)		4 (4)*	3 (6)*	2 (5)*	1 (10)*	-2 (4)*	-5 (4)	-10 (4)	-3 (5)*	-6 (5)
FILQEEYHLNPET	2	616	628	HEL2	33 (3)		-4 (3)*	-1 (3)*	-2 (3)*	-3 (2)*	-1 (3)*	1 (4)*	-2 (3)*	0 (2)*	-4 (2)*
ILQEEYHLNPETITIL	2	617	632		22 (1)		-1 (1)*	1 (1)*	1 (1)*	-3 (1)*	2 (3)*	1 (2)*	-2 (3)*	-1 (2)*	-3 (2)*
EEYHLNPETITILFVKTR	2	620	637		38 (2)		1 (2)*	5 (3)*	2 (5)*	-6 (3)	5 (4)	0 (4)*	3 (3)*	0 (4)*	4 (5)*
FVKTRALVDALKNWIEGNPKL	3	633	653		31 (2)		-3 (3)*	-2 (2)*	-1 (3)*	-8 (3)	-2 (2)*	1 (3)*	-6 (3)	3 (3)*	-11 (2)
FVKTRALVDALKNWIEGNPKLSF	3	633	655		35 (2)		-3 (2)*	-3 (2)*	-2 (2)*	-10 (2)		1 (3)*	-6 (2)	4 (4)*	-10 (2)
FVKTRALVDALKNWIEGNPKLSFLKPGIL	4	633	661	motif IV	36 (2)		-3 (3)*	0 (2)*	5 (2)	-12 (2)	-3 (0)*	2 (5)*	-6 (3)		-12 (4)
SFLKPGIL	2	654	661		40 (2)		1 (2)*	3 (3)*	1 (5)*	-2 (3)*	0 (2)*	-1 (5)*	-8 (2)	-5 (5)	-12 (5)
TGRGKTNQNTGMTLPAQKCIL	3	662	682		72 (4)		3 (5)*	0 (6)*	-4 (5)*	-3 (5)*	-4 (5)*	-3 (4)*	-4 (4)*	-6 (5)	-1 (7)*
TGRGKTNQNTGMTLPAQKCIL	4	662	682		67 (3)		4 (4)*	-3 (4)*	-5 (3)*	-1 (4)*	-2 (5)*	-4 (4)*	-3 (3)*	-6 (5)	0 (0)*
PAQKCIL	2	676	682		85 (3)		-1 (5)*	1 (3)*	0 (4)*	-3 (6)*	-2 (4)*	1 (2)*	-1 (3)*	-14 (6)	-4 (5)*
DAFKASGDHNIL	2	683	694		46 (3)		0 (3)*	2 (4)*	-2 (3)*	-2 (4)*	-1 (4)*	-2 (3)*	-1 (2)*	-4 (3)*	0 (4)*
DAFKASGDHNIL	3	683	694		47 (2)		0 (3)*	0 (4)*	-3 (3)*	-2 (5)*	0 (3)*	-2 (3)*	-2 (3)*	-4 (3)*	1 (4)*
FKASGDHNIL	3	685	694		39 (3)		-1 (3)*	0 (4)*	-3 (3)*	-3 (4)*	1 (5)*	-1 (3)*	0 (3)*	-2 (2)*	0 (4)*
IATSVADEGID	2	695	705	Pincer	71 (2)		0 (4)*	-4 (3)*	-5 (4)	-3 (6)*	0 (6)*	-2 (3)*	-49 (5)	-9 (4)	-39 (5)
IATSVADEGIDIAQCNLVILYEYVGNVIKM	3	695	724		68 (2)		1 (3)*	-1 (3)*	-3 (4)*	-2 (1)*	0 (2)*	-3 (4)*	-38 (4)	-4 (4)*	-24 (3)
YEYVGNVIKM	2	715	724		83 (4)		0 (6)*	-5 (4)*	-2 (4)*	-8 (4)	-2 (6)*	-4 (5)*	-39 (5)	-12 (4)	-40 (3)
YVGNVIKM	2	717	724		72 (3)		2 (5)*	-1 (4)*	2 (4)*	0 (3)*	-1 (6)*	-4 (5)*	-34 (4)	-12 (4)	-35 (3)
IQTRGRGRARGSKC	4	725	738		74 (2)		3 (4)*	-3 (4)*	-3 (7)*	0 (5)*	3 (0)*	-7 (4)	-38 (5)	2 (7)*	-31 (6)
FLLTSNAGVIEKEQINM	2	739	755		44 (3)		-1 (4)*	2 (4)*	0 (5)*	-23 (3)	3 (5)*	2 (4)*	-9 (3)	-4 (3)*	-12 (4)
LTSNAGVIEKEQINM	2	741	755		55 (3)		-3 (5)*	-1 (4)*	-2 (4)*	-7 (3)	-1 (6)*	0 (1)*	-10 (4)	-5 (3)*	-15 (3)
AGVIEKE	2	745	751		51 (3)		4 (4)*	2 (3)*	-3 (5)*		-3 (1)*	-2 (1)*	-7 (3)	-1 (5)*	-2 (5)*
KEQINM	2	750	755		41 (3)		2 (4)*	3 (4)*	2 (5)*	-3 (3)*	-5 (7)*	4 (5)*	-24 (2)	-10 (3)	-26 (6)
YKEKMMNDSIL	2	756	766		18 (2)		4 (3)*	10 (3)	3 (5)*	-1 (3)*	5 (5)	6 (4)	2 (3)*	-1 (3)*	1 (5)*
YKEKMMNDSIL	3	756	766		18 (3)		5 (4)*	6 (3)	3 (5)*	-1 (3)*	5 (4)	6 (4)	1 (3)*	-1 (3)*	1 (5)*
RLQTWDEAVF	2	767	776		52 (2)		-5 (4)*	-1 (3)*	-4 (3)*	1 (4)*	0 (3)*	0 (4)*	5 (2)	-1 (2)*	3 (2)*
WDEAVF	1	771	776		50 (3)		0 (4)*	-4 (2)*	4 (2)*	2 (2)*	1 (4)*	-2 (3)*	0 (4)*	-1 (7)*	1 (5)*
FREKILHIQTHEKF	4	776	789		19 (4)		-6 (4)	1 (4)*	0 (5)*	-7 (4)	-2 (0)*	2 (4)*	4 (5)*	1 (3)*	-4 (5)*
REKILHIQTHEKF	3	777	789		22 (2)		-7 (4)	0 (3)*	0 (5)*	-8 (4)	0 (3)*	3 (4)*	0 (3)*		-6 (5)
RDSQEKPKPVPDK	2	791	803	CTD	81 (4)		1 (4)*	0 (5)*	-2 (4)*	-5 (7)	0 (6)*	-11 (5)	-15 (3)		-18 (3)
VRVIEE	2	823	828		62 (3)		0 (4)*	4 (4)*	0 (4)*	-13 (4)	-3 (4)*	0 (4)*	-3 (7)*	-1 (2)*	-2 (2)*
VRVIEECHY	2	823	831		41 (2)		-6 (4)	3 (3)*	4 (3)*		-3 (4)*	1 (4)*	2 (5)*	-1 (3)*	-2 (3)*
VRVIEECHYTVLGDA	3	823	837		42 (4)		-1 (2)*	1 (2)*	-2 (4)*	-8 (4)	-2 (2)*	-1 (2)*		0 (3)*	0 (5)*
VRVIEECHY	3	823	831		35 (2)		-7 (5)	-2 (4)*	0 (5)*	-9 (5)	-2 (6)*	-1 (4)*	3 (4)*	-2 (3)*	-2 (5)*
CHYTVLGDA	2	829	837		26 (1)		2 (2)*	3 (2)*	1 (2)*	0 (2)*	3 (3)*	-1 (3)*	0 (4)*	0 (2)*	-1 (2)*
TVLGDAFKECF	2	832	842		49 (2)		-3 (3)*	-1 (3)*	-1 (3)*	-6 (3)	-1 (3)*	0 (4)*	0 (3)*	1 (4)*	0 (4)*
FKECF	1	838	842		31 (3)		-8 (3)	-3 (4)*	-2 (5)*	-10 (4)	3 (7)*	1 (5)*	-1 (3)*	5 (3)*	-1 (5)*
FKECF	2	838	842		34 (2)		-9 (3)	-6 (4)	-9 (5)	-9 (4)	-2 (5)*	2 (6)*	0 (5)*	5 (4)*	0 (5)*
FVSRPHPKPKQFSS	4	842	855	Capping loop	77 (4)		-12 (5)	-13 (6)	-4 (4)*	-13 (6)	-21 (5)	1 (5)*	4 (3)*	-3 (6)*	-4 (5)*
VSRPHPKPKQFSSF	3	843	856		74 (5)		-10 (6)	-15 (5)	-4 (5)*	-12 (6)	-24 (4)	0 (5)*	2 (6)*	-3 (6)*	-3 (6)*
VSRPHPKPKQFSSF	4	843	856		77 (4)		-12 (5)	-13 (6)	-2 (4)*	-13 (6)	-22 (6)	1 (5)*	4 (3)*	-3 (6)*	-4 (5)*
EKRAKIF	2	857	863		43 (3)		-5 (4)*	-4 (5)*	-5 (5)*	-11 (4)	-2 (4)*	-1 (6)*	-3 (3)*	-1 (4)*	-5 (5)
EKRAKIFC	2	857	864		40 (3)		-3 (3)*	-1 (5)*	-6 (5)	-9 (6)	0 (4)*	0 (3)*	0 (2)*		-3 (5)*
EKRAKIFC	3	857	864		41 (4)		-6 (4)	-3 (5)*	-4 (6)*	-11 (5)	2 (4)*	0 (5)*	-1 (3)*	0 (4)*	0 (6)*
CARQNCSHDWGIHVKYKT	4	864	881		37 (3)		-1 (3)*	-3 (4)*	-3 (5)*	-7 (4)		0 (5)*	-2 (2)*	2 (3)*	-1 (4)*
FEIPVIKIESF	2	882	892		11 (2)		-7 (2)	-5 (2)*	-4 (3)*	-10 (2)	-1 (4)*	1 (2)*	1 (2)*	-1 (2)*	-1 (3)*
FEIPVIKIES	2	882	891		16 (2)		-8 (2)	-3 (2)*	-2 (2)*	-6 (2)	-1 (5)*	3 (2)*	3 (2)*	-1 (2)*	1 (2)*
EIPVIKIESF	2	883	892		11 (2)		-8 (2)	-4 (2)*	-2 (2)*	-6 (2)		3 (2)*	3 (2)*	-1 (2)*	1 (2)*
VVEDIATGVQTL	1	893	904	RNA binding region	65 (2)		-12 (4)	-9 (3)	-6 (3)	-14 (3)	-5 (3)	-1 (3)*	-3 (4)*	-5 (4)*	-10 (2)
VVEDIATGVQTL	2	893	904		65 (3)		-10 (4)	-14 (3)	-11 (4)	-16 (4)	-6 (3)	3 (3)*	-1 (4)*	-5 (4)*	-10 (3)
IATGVQTL	2	897	904		66 (3)		-8 (5)	-20 (5)	-22 (5)	-18 (4)	-6 (3)	-1 (6)*	-6 (3)	-7 (5)	-6 (4)
YSKWKDFHFEKIPFDPAE	3	905	922		54 (3)		-9 (4)	-6 (3)	-3 (3)*	-5 (4)*	-1 (1)*	2 (4)*	1 (3)*	3 (4)*	-10 (4)
EKIPFDPAE	2	914	922		35 (3)		2 (3)*	1 (3)*	-1 (3)*	-3 (3)*	2 (5)*	0 (4)*	-1 (4)*	-1 (4)*	1 (3)*
EKIPFDPAEMSK	2	914	925		54 (3)		-2 (4)*	-1 (4)*	-3 (4)*	-5 (4)*	-5 (3)	-1 (3)*	-1 (2)*	-2 (5)*	-1 (5)*
EKIPFDPAEMSK	3	914	925		57 (3)		0 (4)*	0 (4)*	-2 (4)*	-5 (4)*	-3 (3)*	-2 (3)*	-2 (3)*	-2 (4)*	-2 (6)*

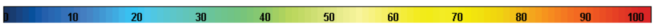

HDX Dynamics Key

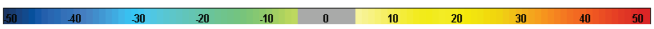

HDX Perturbation Key

H830A RIG-I peptide sequence	charge	start	end	structure	(xii)	(xiii)	(xiv)
FQDYIRKTLDPTYILS	3	12	27		52 (3)	11 (3)	11 (3)
QDYIRKTLDPTYIL	3	13	26		78 (3)	11 (3)	10 (5)
IRKTLDPTYIL	3	16	26		46 (3)	17 (4)	16 (6)
SYMAPWFRE	2	27	35		53 (3)	14 (4)	14 (4)
YMAPWFREEEVQ	2	28	39	CARD1	55 (3)	14 (4)	14 (4)
VQYIQA	2	38	43		66 (4)	8 (5)	8 (5)
YIQAEKNNKGPMEA	3	40	53		51 (3)	9 (4)	8 (4)
EKNNKGPMEAATL	2	44	56		45 (2)	11 (3)	11 (5)
LQEEGWFRGFL	2	64	74		48 (2)	9 (4)	6 (6)
QEEGWFRGFL	2	65	74		52 (1)	9 (3)	8 (4)
FRGFLDA	2	70	76		16 (2)	20 (3)	19 (4)
DALDHAGYSGL	2	75	85		34 (2)	13 (3)	13 (3)
LDHAGYSGL	2	77	85		36 (2)	10 (3)	9 (3)
YEAIESW	1	86	92		35 (2)	25 (5)	23 (4)
YEAIESWD	1	86	93		29 (3)	23 (5)	22 (5)
YRLLL	2	103	107		12 (3)	24 (5)	23 (5)
YRLLLKRLQPEF	3	103	114	Latch region	17 (3)	33 (5)	33 (5)
LLKRLQPEF	2	106	114		21 (4)	30 (5)	28 (6)
LLKRLQPEF	3	106	114		18 (4)	33 (5)	34 (6)
LLKRLQPEFKTRIIPTDIISD	4	106	126		7 (1)	10 (1)	8 (2)
KRLQPEF	2	108	114		21 (4)	34 (5)	33 (6)
KRLQPEF	3	108	114		18 (4)	33 (4)	33 (6)
KTRIIPTDIISD	3	115	126		62 (2)	12 (4)	11 (4)
KTRIIPTDIISDL	2	115	127	CARD2	59 (2)	14 (4)	13 (4)
KTRIIPTDIISDL	3	115	127		58 (3)	14 (4)	13 (4)
KTRIIPTDIISDLSE	3	115	129		63 (2)	11 (4)	11 (4)
EEILQICSTKGMMAGAEKL	3	137	155		31 (1)	17 (3)	8 (5)
ILQICSTKGMMAGA	2	139	152		40 (2)	20 (2)	20 (3)
ILQICSTKGMMAGAEKL	3	139	155		37 (2)	16 (3)	15 (4)
ILQICSTKGMMAGAEKLVE	3	139	157		29 (1)	19 (2)	18 (3)
LLRSDKENWPKTLKL	4	159	173		29 (2)	10 (3)	18 (3)
RSDKENWPKTLKL	3	161	173		28 (2)	13 (4)	10 (6)
ALEKERNKFSEL	4	174	185		67 (1)	15 (5)	13 (8)
ALEKERNKFSEL	3	174	185		61 (3)	8 (3)	8 (4)
EKERNKFSEL	3	176	185		65 (2)	10 (4)	10 (6)
WIVEKGIKDVE	3	186	196	linker	102 (3)	0 (5)*	0 (6)*
WIVEKGIKDVETEDL	3	186	200		98 (2)	-2 (5)*	2 (3)*
WIVEKGIKDVETEDLED	2	186	202		97 (3)	0 (4)*	1 (5)*
WIVEKGIKDVETEDLEDKMETSD	3	186	208		95 (2)	0 (3)*	-1 (6)*
IVEKGIKDVETEDL	3	187	200		95 (5)	-2 (5)*	1 (5)*
EDKMETSDIQIF	2	201	212		84 (3)	1 (4)*	1 (6)*
IQIFYQEDPECQNLSE	2	209	224		70 (2)	0 (3)*	-2 (6)*
FYQEDPECQNL	2	212	222		81 (2)	-2 (3)*	-2 (6)*
YQEDPECQNL	2	213	222		81 (3)	-1 (4)*	-3 (6)*
SENSCPPSEVSDTNL	2	223	237		83 (4)	-2 (6)*	-1 (4)*
NSCPPSEVSDTNL	2	225	237		82 (4)	-1 (6)*	-1 (3)*
VSDTNL	1	232	237		92 (5)	-2 (6)*	-2 (3)*
YSPFKPRN	3	238	245		100 (4)	-2 (5)*	-1 (7)*
YSPFKPRNYQLE	3	238	249		74 (3)	4 (4)*	3 (5)*
YSPFKPRNYQLE	2	238	249		73 (3)	3 (4)*	3 (5)*
LALPAMKGKNTIIC	2	250	263	HEL1	32 (3)	-1 (4)*	1 (5)*
LALPAMKGKNTIICAPTGCGKTFVSLL	4	250	276		47 (2)	4 (3)*	2 (5)*
LALPAMKGKNTIICAPTGCGKTFVSLL	3	250	276		43 (2)	-1 (3)*	-5 (3)
APTGCGKTF	2	264	272		84 (3)	-1 (5)*	-1 (6)*
LICEHHLKKFPQGQKGKVVF	3	276	295		29 (1)	0 (2)*	0 (3)*
ICEHHLKKFPQGQKGKVVF	3	277	295		28 (2)	-3 (2)*	-3 (4)*
PQGQKGKVVF	3	286	295		79 (4)	-1 (4)*	-1 (7)*
FANQIPVYEQQ	2	296	306		74 (2)	-18 (3)	-15 (4)
FANQIPVYEQQKSVF	2	296	310	motif Ia	66 (2)	-16 (4)	-14 (5)
FANQIPVYEQQKSVFSKY	3	296	313		56 (1)	-10 (2)	-11 (3)
FERHGYRVTGISGATAENVPVEQIVENND	4	314	342		66 (3)	-4 (4)*	-3 (4)*
FERHGYRVTGISGATAENVPVEQIVENND	3	314	342		65 (3)	-4 (4)*	0 (7)*
GYRVTGISGATAENVPVEQIVENN	4	318	341		71 (2)	-3 (5)*	-5 (6)*
YRVTGISGATAENVPVEQIVE	3	319	339		84 (5)	-2 (7)*	-2 (8)*
IIILTPQIL	2	343	351		38 (1)	-5 (2)*	-4 (2)*
IIILTPQIL	1	343	351		35 (1)	-4 (2)*	-3 (2)*
IIILTPQILVN	2	343	353		38 (1)	-6 (2)	-5 (2)*
IIILTPQILVNN	2	343	354		43 (2)	-8 (3)	-8 (4)
IIILTPQILVNNL	2	343	355		52 (1)	-11 (2)	-10 (3)
IIILTPQILVNNLKKGTIPSL	3	343	363		72 (3)	-10 (3)	-8 (4)
IIILTPQILVNNLKKGTIPSL	2	343	363	motif Ic	68 (2)	-7 (4)	-6 (4)
IIILTPQILVNNLKKGTIPSLSI	3	343	365		68 (2)	-9 (3)	-7 (4)
IIILTPQILVNNLKKGTIPSLSIF	3	343	366		65 (2)	-9 (3)	-7 (3)
KGTIPSLSIFT	2	357	367		61 (1)	4 (3)*	4 (3)*
KGTIPSLSIFT	3	357	367		61 (1)	4 (3)*	4 (3)*
LMIFDECHNTSKQHPYNM	3	368	385		36 (1)	-6 (2)	-6 (2)
IFDECHNTSKQHPYNMIM	3	370	387	motif IIa	36 (2)	-6 (2)	-6 (2)
IFDECHNTSKQHPYNMIM	2	370	387		36 (1)	-6 (2)	-6 (3)
FNYLDQKLGGSSGPLPQVIG	2	388	407		37 (1)	0 (2)*	0 (3)*
FNYLDQKLGGSSGPLPQVIGL	2	388	408		36 (1)	0 (2)*	0 (3)*
DQKLGGSSGPLPQVIGL	2	392	408		43 (1)	0 (2)*	0 (3)*
LTASVGVGDAKNTDEAL	2	408	424		70 (3)	-5 (4)	-5 (5)*
TASVGVGDAKNTDEAL	2	409	424		72 (3)	-5 (4)	-5 (5)*
TASVGVGDAKNTDEALD	2	409	425		69 (3)	-5 (4)*	-4 (5)*
DYICKL	2	425	430		3 (1)	0 (1)*	1 (1)*
DYICKL	1	425	430		3 (0)	0 (2)*	1 (2)*
CASLDASVIATVKHNLEEL	2	431	449		37 (1)	0 (2)*	0 (3)*
SVIATVKHNLEE	2	437	448		62 (2)	-1 (3)*	-1 (3)*
EQVVYKPQKF	3	450	459		76 (2)	1 (4)*	1 (4)*
VVYKPQKF	2	452	459		83 (3)	-1 (5)*	-1 (5)*
VVYKPQKF	3	452	459		83 (3)	-1 (4)*	-1 (5)*
FRKVESRISDKF	3	460	471		71 (2)	0 (3)*	-3 (16)*
QLMRDTESL	2	477	485	HEL2i	9 (2)	0 (2)*	-1 (5)*
MRDTESL	2	479	485		7 (0)	1 (1)*	1 (1)*
AKRICKDLENL	3	486	496		76 (3)	-8 (3)	-6 (5)
SQIQNREFGTQKYEQ	3	497	511		76 (3)	-4 (5)*	-4 (5)*
SQIQNREFGTQKYEQW	3	497	512		70 (3)	-3 (4)*	-3 (5)*
WIVTVQKACM	2	512	521		11 (4)	0 (6)*	0 (5)*
IVTVQKACM	2	513	521		12 (1)	-2 (2)*	-1 (2)*
VFQMPDKDEESRICKA	3	522	537		66 (2)	-11 (3)	-11 (4)
VFQMPDKDEESRICKALF	4	522	539	CTD:HEL2i	58 (2)	-10 (3)	-9 (3)
FQMPDKDEESRICKA	3	523	537		64 (2)	-11 (3)	-11 (4)
FQMPDKDEESRICKALF	4	523	539		53 (2)	-9 (2)	-9 (3)
LYTSHLRKYNDAL	4	540	552		8 (2)	2 (4)*	2 (4)*
LYTSHLRKYNDAL	3	540	552		8 (2)	2 (3)*	2 (4)*

H830A RIG-I

peptide sequence	charge	start	end	structure	(xii)	(xiii)	(xiv)
LYTSHLRKYNDAL	4	540	552	HEL2i	8 (2)	2 (4)*	2 (4)*
LYTSHLRKYNDAL	3	540	552		8 (2)	2 (3)*	2 (4)*
IISEHARMKDALD	3	553	565		23 (2)	2 (3)*	2 (3)*
HARMKDALD	2	557	565		32 (2)	1 (2)*	1 (3)*
HARMKDALD	3	557	565		31 (2)	1 (2)*	1 (3)*
DYLKDFFSNVRAAGF	3	565	579		49 (2)	14 (4)	12 (4)
YLKDFFSNV	2	566	574	Gate motif	18 (1)	14 (2)	13 (2)
YLKDFFSNVRAAGF	3	566	579		46 (5)	12 (5)	12 (3)
FFSNV	1	570	574		36 (2)	27 (4)	25 (4)
FFSNVRAAGF	2	570	579		58 (2)	19 (5)	17 (5)
FFSNVRAAGFD	2	570	580		63 (5)	15 (6)	14 (6)
FSNVRAAGF	2	571	579		65 (2)	19 (4)	17 (5)
DEIEQDLTQRFEEKLQE	3	580	596		25 (0)	5 (2)	6 (2)
DEIEQDLTQRFEEKLQEL	3	580	597		25 (1)	6 (2)	6 (2)
DEIEQDLTQRFEEKLQELES	3	580	599		28 (1)	5 (2)	6 (2)
QELESVSRDPSNENPKLED	3	595	613		53 (2)	0 (3)*	0 (4)*
ESVSRDPSNENPKLEDLC	3	598	615		45 (1)	0 (2)*	0 (2)*
VSRDPSNENPKL	3	600	611		68 (4)	0 (6)*	-2 (6)*
VSRDPSNENPKLED	2	600	613		58 (3)	1 (4)*	0 (5)*
FILQEEYHLNPET	2	616	628	HEL2	31 (1)	0 (2)*	0 (2)*
ILQEEYHLNPETITIL	2	617	632		26 (1)	0 (1)*	0 (2)*
EEYHLNPETITILFVKTR	2	620	637		40 (1)	0 (2)*	1 (3)*
FVKTRALVDALKNWIEGNPKL	3	633	653	motif IV	30 (1)	-1 (2)*	-2 (3)*
FVKTRALVDALKNWIEGNPKLSF	3	633	655		32 (1)	-1 (3)*	-3 (4)*
FVKTRALVDALKNWIEGNPKLSFLKPGIL	4	633	661		33 (1)	0 (2)*	-2 (5)*
WIEGNPKL	2	646	653		42 (1)	0 (2)*	1 (3)*
WIEGNPKLSFLKPGIL	2	646	661		44 (2)	4 (2)*	1 (4)*
SFLKPGIL	2	654	661		43 (2)	1 (2)*	2 (4)*
KPGILTGRGKTNQNTG	3	657	672		30 (4)	-4 (5)*	-1 (5)*
TGRGKTNQNTGMTLPAQKCIL	3	662	682		84 (3)	-3 (4)*	-3 (5)*
PAQKCIL	2	676	682		77 (1)	-4 (2)*	3 (6)*
DAFKASGDHNIL	3	683	694		55 (3)	-1 (4)*	-1 (4)*
DAFKASGDHNIL	2	683	694		52 (3)	0 (4)*	0 (5)*
FKASGDHNIL	3	685	694		46 (3)	0 (4)*	-1 (4)*
IATSVADEGID	2	695	705	Pincer	84 (3)	-5 (5)*	-3 (4)*
IATSVADEGIDIAQCNL	2	695	711		85 (3)	-2 (6)*	0 (5)*
SVADEGIDIAQ	2	698	708		38 (2)	4 (3)*	4 (4)*
CNLVILYEYVGNV	4	709	721		23 (2)	1 (3)*	1 (4)*
VILYEYVGNVIKM	2	712	724		72 (3)	-2 (5)*	0 (4)*
YEYVGNVIKM	2	715	724		97 (3)	-5 (6)*	-5 (6)*
YVGNVIKM	2	717	724		91 (3)	-4 (5)*	-4 (6)*
IQTRGRGRARGSKC	4	725	738		66 (3)	-4 (4)*	-4 (5)*
FLLTSNAGVIE	2	739	749		65 (1)	-5 (2)*	-2 (3)*
FLLTSNAGVIEKEQINM	2	739	755		46 (3)	4 (4)*	1 (4)*
LTSNAGVIEKEQINM	2	741	755		60 (2)	3 (5)*	0 (3)*
AGVIEKE	2	745	751		62 (2)	0 (4)*	0 (6)*
KEQINM	2	750	755		59 (2)	4 (3)*	3 (3)*
YKEKMMNDSIL	3	756	766		21 (2)	11 (3)	11 (4)
YKEKMMNDSIL	2	756	766		21 (2)	11 (3)	11 (4)
SILRLQT	2	764	770		26 (2)	1 (3)*	2 (4)*
RLQTWDEAVF	2	767	776		51 (1)	-2 (3)*	0 (3)*
WDEAVF	1	771	776		75 (2)	0 (3)*	-1 (3)*
FREKILHIQTHEKF	4	776	789		-18 (1)	-2 (2)*	-1 (3)*
REKILHIQTHEKF	3	777	789		9 (2)	0 (4)*	-2 (3)*
THEKFIRDS	2	785	793	CTD	18 (1)	14 (2)	13 (2)
EKFIRDSQEK	2	787	796		63 (2)	10 (4)	4 (4)*
RDSQEKPKPVPDK	2	791	803		98 (2)	0 (4)*	0 (5)*
VRVIEE	2	823	828		65 (2)	-4 (3)*	-4 (3)*
TVLGDAFKECF	2	832	842		56 (2)	-5 (3)*	-3 (3)*
FKECF	1	838	842		46 (2)	-14 (3)	-10 (4)
FKECF	2	838	842		44 (2)	-14 (3)	-10 (4)
FVSRPHPKPKQFSS	4	842	855	Capping loop	77 (2)	-19 (4)	-3 (4)*
VSRPHPKPKQFSSF	4	843	856		78 (3)	-19 (4)	-3 (4)*
VSRPHPKPKQFSSF	3	843	856		77 (2)	-22 (4)	-4 (4)*
EKRAKIF	2	857	863		49 (2)	-14 (2)	-9 (3)
EKRAKIFC	3	857	864		44 (3)	-13 (3)	-10 (3)
EKRAKIFC	2	857	864		43 (2)	-13 (4)	-8 (3)
CARQNCSHDWGIHVKYKT	4	864	881		15 (2)	-3 (2)*	-2 (2)*
DWGIHVKYKTFEIPVIKIESFVV	4	872	894		47 (2)	4 (3)*	3 (5)*
FEIPVIKIESF	2	882	892		14 (1)	-5 (3)	-5 (3)*
FVVEDIATGVQTL	2	892	904	RNA binding region	64 (2)	-12 (4)	-11 (5)
VVEDIATGVQTL	2	893	904		74 (2)	-14 (3)	-12 (4)
VVEDIATGVQTL	1	893	904		72 (2)	-15 (3)	-12 (4)
YSKWKDFHFEKIPFDPAE	3	905	922		52 (2)	-4 (2)*	-4 (6)*
EKIPFDPAE	2	914	922		46 (3)	1 (3)*	1 (4)*
EKIPFDPAEMSK	2	914	925		65 (4)	-2 (5)*	-3 (6)*
EKIPFDPAEMSK	3	914	925		66 (4)	-1 (5)*	-2 (6)*

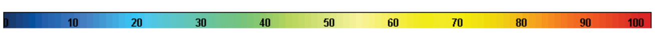

HDX Dynamics Key

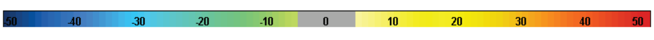

HDX Perturbation Key

E373A RIG-I

peptide sequence	charge	start	end	structure	(xv)	(xvi)	(xvii)	(xviii)	(xix)	(xx)	(xxi)	(xxii)
FQDYIRKTLDPTYIL	2	12	26		34 (2)	14 (3)	6 (3)	0 (3)*	2 (2)*	1 (2)*	7 (3)	13 (3)
FQDYIRKTLDPTYIL	3	12	26		33 (2)	14 (3)	6 (3)	0 (3)*	2 (2)*	1 (2)*	7 (3)	13 (3)
FQDYIRKTLDPTYILS	3	12	27		49 (1)	10 (1)	3 (2)*	0 (1)*	3 (1)*	2 (3)*	7 (4)	24 (4)
SYMAPWFREEEVQ	2	27	39		45 (2)	15 (3)	7 (3)	0 (2)*	4 (3)*	2 (3)*	8 (2)	12 (3)
YMAPWFRE	2	28	35		52 (2)	12 (3)	5 (3)*	-1 (2)*	4 (3)*	-1 (3)*	8 (3)	10 (4)
YMAPWFREEE	2	28	37	CARD1	51 (2)	13 (3)	6 (3)	1 (2)*	3 (4)*	1 (4)*	7 (3)	8 (4)
YMAPWFREEEVQ	2	28	39		47 (2)	15 (3)	7 (3)	0 (2)*	4 (3)*	1 (4)*	8 (2)	11 (3)
VQYIQA	1	38	43		48 (2)	16 (3)	6 (3)	-3 (3)*	3 (2)*	2 (3)*	8 (3)	16 (4)
YIQAEKNNKGPMEAATL	3	40	56		38 (2)	14 (3)	7 (3)	0 (3)*	4 (3)*	3 (3)*	8 (3)	13 (3)
LQEEGWFRGFL	2	64	74		38 (1)	17 (1)	13 (2)	1 (2)*	1 (4)*	-1 (3)*	1 (4)*	14 (3)
QEEGWFRGFL	2	65	74		47 (2)	8 (2)	4 (3)*	2 (2)*	3 (3)*	0 (3)*	4 (3)*	6 (3)
QEEGWFRGFLDAL	2	65	77		45 (3)	21 (3)	10 (4)	0 (4)*	3 (3)*	0 (4)*	8 (3)	14 (4)
FRGFL	2	70	74		19 (1)	14 (2)	6 (2)	-2 (2)*	3 (1)*	0 (2)*	7 (2)	13 (2)
FRGFLDA	2	70	76		15 (1)	15 (2)	7 (2)	-2 (1)*	3 (1)*	1 (1)*	7 (2)	13 (2)
DALDHAGYSGL	2	75	85		30 (2)	12 (2)	5 (2)	0 (3)*	3 (2)*	2 (3)*	7 (2)	10 (3)
LDHAGYSGL	2	77	85		34 (2)	8 (2)	5 (3)*	0 (2)*	5 (2)	5 (3)*	8 (3)	9 (3)
YEAIESWD	1	86	93		28 (2)	18 (2)	7 (2)	-3 (2)*	4 (3)*	2 (2)*	9 (3)	17 (2)
YEAIESWDFKKIEKLEE	3	86	102		52 (2)	7 (2)	0 (3)*	-3 (3)*	0 (2)*	-5 (5)*	0 (4)*	
EAIESWDFKKIEKLEEY	4	87	103		55 (3)	10 (3)	5 (3)*	2 (2)*	2 (3)*		4 (4)*	6 (4)
DFKKIEKLEE	3	93	102		69 (2)	6 (3)	2 (3)*	1 (2)*	1 (5)*	-2 (6)*	1 (4)*	3 (4)*
FKKIEKLEE	3	94	102		63 (2)	5 (3)	2 (3)*	1 (2)*	-2 (4)*	-3 (4)*	1 (3)*	3 (4)*
YRLLL	2	103	107		11 (4)	19 (4)	8 (5)	0 (4)*	2 (2)*	0 (2)*	8 (5)	15 (3)
YRLLLKRLQPEF	3	103	114		15 (3)	32 (4)	9 (5)	3 (3)*	-1 (2)*	5 (2)*	14 (4)	14 (4)
LLKRLQPEF	2	106	114		19 (4)	26 (5)	13 (5)	3 (5)*	4 (2)*	7 (2)	15 (3)	22 (3)
KRLQPEF	2	108	114	Latch region	19 (5)	31 (5)	15 (6)	5 (6)*	2 (2)*	7 (3)	23 (4)	24 (4)
KRLQPEF	3	108	114		15 (5)	31 (5)	16 (6)	0 (6)*	4 (2)*	2 (3)*	13 (4)	22 (4)
KTRIIPTD	2	115	122		53 (3)	15 (5)	8 (4)	2 (4)*	1 (5)*	-1 (6)*	5 (4)	8 (5)
KTRIIPTDIISD	3	115	126		54 (2)	13 (4)	8 (4)	-1 (3)*	3 (4)*	0 (5)*	5 (4)	7 (4)
KTRIIPTDIISDL	2	115	127		52 (2)	14 (3)	8 (3)	2 (2)*	2 (4)*	1 (4)*	6 (3)	9 (4)
KTRIIPTDIISDL	3	115	127		51 (2)	14 (3)	8 (3)	1 (2)*	2 (4)*	1 (4)*	6 (3)	9 (4)
KTRIIPTDIISDLSE	3	115	129		56 (2)	15 (4)	11 (3)	-1 (2)*	2 (3)*	3 (4)*	12 (3)	8 (3)
ILQICSTKGMMAGAEKL	3	139	155	CARD2	34 (2)	16 (2)	8 (2)	3 (3)*	3 (3)*	0 (2)*	5 (4)	12 (3)
ILQICSTKGMMAGAEKLVE	3	139	157		27 (2)	20 (3)	10 (2)	3 (2)*	4 (2)*	1 (2)*	8 (2)	14 (3)
ICSTKGMMAGAEKL	3	142	155		34 (2)	21 (3)	12 (3)	3 (3)*	4 (3)*	1 (3)*	8 (3)	13 (3)
LRSDKENWPKTLKL	4	160	173		29 (3)	20 (3)	8 (3)	2 (3)*	-6 (3)	-6 (4)	2 (4)*	11 (2)
LRSDKENWPKTLKLAL	4	160	175		26 (3)	21 (3)	8 (4)	4 (3)*	-4 (3)*	-8 (3)	3 (4)*	6 (2)
ALEKERNKFSEL	3	174	185		62 (2)	10 (4)	12 (4)	1 (2)*	1 (4)*	1 (4)*	-1 (6)*	4 (5)*
EKERNKFSEL	3	176	185		59 (1)	10 (4)	6 (3)	1 (2)*	-9 (4)	-3 (5)*	-2 (4)*	4 (4)*
LWIVEKGIKDVETED	3	185	199		88 (2)	2 (4)*	3 (6)*	1 (5)*	0 (2)*	-1 (4)*	1 (5)*	-1 (6)*
WIVEKGIKD	3	186	194		76 (3)	3 (5)*	4 (5)*	1 (4)*	4 (4)*	2 (3)*	3 (5)*	1 (5)*
WIVEKGIKDVE	2	186	196		89 (1)	2 (4)*	3 (5)*	2 (4)*	1 (3)*	-1 (3)*	1 (3)*	-1 (6)*
WIVEKGIKDVE	3	186	196		89 (1)	2 (4)*	4 (5)*	2 (4)*	1 (2)*	0 (9)*	2 (5)*	0 (6)*
WIVEKGIKDVETEDLED	2	186	202		85 (3)	2 (5)*	3 (6)*	4 (5)*	1 (5)*	-1 (5)*	1 (5)*	-2 (5)*
EDKMETSD	2	201	208		75 (1)	1 (4)*	1 (1)*	0 (4)*	1 (1)*	0 (3)*	0 (1)*	-5 (1)*
IQIFYQEDPECQNL	2	209	222	linker	82 (2)	4 (5)*	5 (5)*	-7 (4)	1 (5)*	1 (5)*	2 (6)*	-2 (5)*
YQEDPECQNL	2	213	222		77 (3)	1 (5)*	2 (6)*	-10 (5)	1 (4)*	0 (6)*	2 (6)*	-1 (7)*
YQEDPECQNLSE	2	213	224		76 (3)	1 (5)*	2 (6)*	-9 (5)	0 (6)*	0 (6)*	2 (6)*	0 (5)*
SENSCPPSEVSDTNL	2	223	237		77 (3)	0 (5)*	2 (6)*	-9 (6)	1 (4)*	0 (5)*	2 (6)*	0 (7)*
YSPFKPRN	2	238	245		89 (2)	2 (6)*	2 (6)*	1 (5)*	-5 (4)	-8 (4)	-5 (7)	-10 (6)
YSPFKPRN	3	238	245		89 (2)	2 (6)*	2 (6)*	1 (5)*	-3 (4)*	-8 (6)	-4 (5)*	-8 (6)
YSPFKPRNYQLE	2	238	249		60 (2)	7 (4)	5 (4)	2 (4)*	-4 (4)*	-9 (5)	-3 (4)*	-9 (4)
LALPAMKGKN	3	250	259		45 (4)	3 (5)*	2 (6)*	3 (6)*	2 (4)*	0 (5)*	2 (5)*	2 (5)*
LALPAMKGKNTIIC	3	250	263		32 (4)	2 (5)*	1 (5)*	3 (5)*	-2 (4)*	-4 (5)*	0 (5)*	-2 (5)*
LALPAMKGKNTIICA	3	250	264		67 (2)	4 (3)*	4 (3)*	1 (4)*	-1 (4)*	-4 (5)*	0 (5)*	-5 (4)*
LALPAMKGKNTIICAPTGCGKT	4	250	271		52 (3)	-3 (4)*	-1 (4)*	-6 (3)	-5 (3)*	-17 (3)	-9 (4)	-24 (4)
LALPAMKGKNTIICAPTGCGKTFVSL	3	250	275		46 (2)	3 (3)*	2 (4)*	-2 (4)*	-5 (3)	-12 (3)	-6 (4)	-17 (4)
LALPAMKGKNTIICAPTGCGKTFVSL	4	250	275	HEL1	43 (2)	2 (3)*	-1 (3)*	-6 (3)	-2 (3)*	-9 (3)	-5 (2)*	-11 (3)
LICEHHLKKFPQGQKGKVVF	4	276	295		32 (2)	1 (3)*	-3 (3)*	-4 (2)*	-15 (3)	-23 (3)	-10 (5)	-23 (3)
ICEHHLKKFPQGQKGKVVF	4	277	295		33 (2)	0 (3)*	-6 (3)	-10 (3)	-15 (3)	-28 (3)	-11 (5)	-25 (3)
FANQIPVYEQQKSVF	2	296	310		65 (2)	-16 (3)	-6 (4)	-10 (4)	2 (3)*	-5 (4)	-3 (4)*	-29 (4)
FANQIPVYEQQKSVFSKY	3	296	313		57 (2)	-14 (3)	-8 (3)	-3 (4)*	2 (3)*	-6 (3)	3 (3)*	-21 (4)
FERHGYRVTGISGATAENVPVEQ	3	314	336		66 (2)	0 (4)*	0 (4)*	-2 (3)*	-2 (4)*	-2 (3)*	-1 (5)*	-3 (4)*
FERHGYRVTGISGATAENVPVEQ	4	314	336		71 (5)	2 (5)*	1 (5)*	-3 (5)*	2 (3)*	-7 (4)	1 (3)*	-3 (4)*
IIILTPQIL	2	343	351		32 (2)	-2 (3)*	0 (3)*	-10 (2)	-1 (2)*	-3 (2)*	-1 (3)*	-13 (3)
IIILTPQILVNNL	2	343	355		47 (2)	-8 (3)	-4 (3)*	-10 (3)	-2 (2)*	-6 (2)	1 (3)*	-23 (3)
IIILTPQILVNNLKKGTIPSL	3	343	363		61 (2)	-6 (3)	-2 (3)*	-6 (3)	-2 (4)*	-5 (4)*	-2 (3)*	-16 (3)
IIILTPQILVNNLKKGTIPSLSIF	3	343	366	motif Ia	59 (2)	-6 (3)	-2 (3)*	-11 (4)	-2 (3)*	-5 (3)*	-2 (3)*	-14 (3)
VNNLKKGTIPSL	2	352	363		71 (2)	-7 (4)	-3 (4)*	-5 (4)*	-2 (5)*	-5 (5)	-3 (4)*	-11 (4)
VNNLKKGTIPSL	3	352	363		71 (2)	-7 (4)	-3 (4)*	-5 (4)	-2 (4)*	-5 (5)	-3 (4)*	-11 (4)
IFDACHNTSKQHPYNM	4	370	385		40 (1)	-5 (2)*	0 (3)*	-2 (2)*	0 (2)*	-2 (3)*	-1 (3)*	-15 (3)
IFDACHNTSKQHPYNMIM	3	370	387		34 (1)	-4 (2)*	0 (2)*	-1 (2)*	1 (2)*	-2 (2)*	0 (3)*	-13 (3)
IFDACHNTSKQHPYNMIM	4	370	387		34 (1)	-4 (2)*	1 (2)*	-1 (2)*	2 (2)*	-1 (2)*	0 (2)*	-13 (3)
FNYLDQKLGGSSGPLPQVIG	2	388	407		34 (2)	1 (3)*	1 (4)*	0 (3)*	1 (2)*	0 (3)*	3 (3)*	2 (3)*
DQKLGGSSGPLPQVIG	2	392	407		42 (2)	2 (3)*	0 (3)*	2 (2)*	2 (3)*	1 (4)*	4 (3)*	6 (3)
DQKLGGSSGPLPQVIGL	2	392	408		39 (2)	2 (3)*	1 (4)*	0 (3)*	0 (2)*	0 (3)*	3 (3)*	2 (3)*
TASVGVGDAKNTD	2	409	421		77 (3)	-1 (6)*	2 (12)*		-2 (7)*	-6 (8)	-3 (5)*	-15 (5)
TASVGVGDAKNTDEAL	2	409	424	motif Ic	64 (3)	1 (5)*	1 (4)*	-1 (4)*	-2 (5)*	-6 (6)	-2 (4)*	-12 (4)
TASVGVGDAKNTDEALD	2	409	425		61 (3)	1 (5)*	1 (4)*	-1 (4)*	-1 (5)*	-6 (5)	-2 (4)*	-11 (4)
DYICKL	2	425	430		3 (3)	1 (3)*	0 (4)*	-1 (3)*	0 (2)*	-1 (2)*	1 (4)*	-1 (3)*
YICKL	2	426	430	motif IIa	4 (3)	1 (4)*	1 (5)*	-1 (3)*	0 (2)*	-1 (2)*	1 (5)*	-1 (4)*
CASLDASVIATVKHNLEEL	2	431	449		39 (1)	4 (2)*	2 (2)*	1 (2)*	-3 (3)*	-11 (2)	-3 (2)*	3 (2)*
SVIATVKHNLEE	2	437	448		54 (2)	3 (3)*	2 (3)*	0 (2)*	-3 (4)*	-5 (3)*	-2 (3)*	-4 (3)*
SVIATVKHNLEEL	2	437	449		54 (2)	5 (3)*	3 (3)*	2 (2)*	-6 (4)	-8 (3)	-5 (3)*	-7 (3)
LEQVVYKPQKF	3	449	459		63 (2)	10 (4)	6 (3)	0 (4)*	-9 (4)	-16 (4)	-11 (3)	-17 (3)
EQVVYKPQKF	2	450	459		53 (2)	16 (4)	13 (4)	3 (2)*	-12 (5)	-10 (5)	-6 (3)	-10 (4)
EQVVYKPQKF	3	450	459		62 (2)	12 (4)	8 (3)	0 (3)*	-7 (5)	-14 (5)	-9 (3)	-14 (4)
LMRDTESLAKRICKDLEN	4	478	495		44 (2)	0 (2)*	1 (2)*	-1 (3)*	-7 (2)	-12 (1)	-16 (2)	
MRDTESLAKRICKDLENL	4	479	496		44 (2)	0 (2)*	1 (2)*	-1 (3)*	-6 (2)	-12 (1)	-16 (2)	-6 (2)
AKRICKDLENL	3	486	496	HEL2i	69 (3)	0 (4)*	1 (4)*	1 (3)*	-4 (5)*	-4 (3)*	-3 (4)*	-6 (4)
SQIQNREFGTQKYEQ	3	497	511		70 (2)	0 (5)*	1 (4)*	0 (4)*	0 (5)*	-2 (6)*	1 (4)*	-3 (5)*
SQIQNREFGTQKYEQW	3	497	512		64 (2)	0 (4)*	2 (4)*	0 (3)*	0 (4)*	-2 (5)*	1 (4)*	-4 (4)*
WIVTVQKACM	2	512	521		49 (4)	-4 (9)*	1 (6)*	3 (2)*	-2 (4)*	-1 (4)*	-1 (2)*	-4 (2)*
VFQMPDKDEE	2	522	531		70 (3)	-1 (6)*	0 (5)*	0 (4)*	-1 (5)*	-4 (5)*	-3 (5)*	-18 (5)
VFQMPDKDEESRICKA	3	522	537	CTD:HEL2i	61 (2)	-4 (4)*	-1 (4)*	1 (3)*	-1 (4)*	-5 (4)	-3 (3)*	-22 (3)
VFQMPDKDEESRICKA	4	522	537		61 (2)	-4 (4)*	-1 (3)*	0 (3)*	-2 (4)*	-6 (4)	-4 (3)*	-23 (3)
VFQMPDKDEESRICKALF	3	522	539		54 (1)	-4 (3)*	-1 (3)*	1 (2)*	-2 (3)*	-5 (3)	-3 (3)*	-19 (3)
SRICKALF	3	532	539		26 (2)	-5 (2)*	-2 (2)*	1 (2)*	-3 (2)*	-5 (2)	-4 (3)*	-16 (3)
YTSHLRKYNDALI	3	541	553		7 (2)	2 (4)*	2 (4)*	3 (4)*	0 (4)*	-1 (4)*	0 (4)*	-1 (4)*
IISEHARMKDAL	3	553	564		25 (2)	3 (3)*	1 (4)*	1 (2)*	-2 (2)*	-5 (3)*	-1 (4)*	-3 (4)*
IISEHARMKDALD	3	553	565		22 (2)	2 (3)*	3 (3)*	1 (2)*	0 (3)*	-2 (3)*	0 (4)*	-2 (3)*
DYLKDFFSNVRAAGF	3	565	579		44 (2)	11 (3)	12 (3)	-1 (3)*	-3 (2)*	-3 (3)*	2 (3)*	-5 (4)*
YLKDFFSNV	2	566	574	Gate motif	19 (2)	11 (2)	8 (3)	3 (2)*	1 (2)*	0 (2)*	2 (3)*	2 (3)*
YLKDFFSNVRAAGF	3	566	579		43 (2)	15 (3)	13 (2)	-1 (4)*		3 (4)*	11 (3)	4 (3)*
FFSNVRAAGF	2	570	579		55 (2)	17 (4)	12 (3)	3 (3)*	3 (6)*	0 (6)*	9 (3)	5 (5)*
FDEIEQDLTQRF	2	579	590		21 (2)	7 (2)	4 (3)*	2 (2)*	0 (2)*	-3 (2)*	-3 (3)*	-3 (2)*
FDEIEQDLTQRFEEKL	3	579	594		15 (1)	5 (2)	2 (2)*	5 (2)*	2 (3)*	4 (3)*	5 (2)	1 (2)*
DEIEQDLTQRFEEKLQEL	3	580	597		24 (2)	5 (2)*	2 (2)*	2 (1)*	1 (2)*	-1 (2)*	0 (2)*	-1 (3)*
DEIEQDLTQRFEEKLQELES	3	580	599		26 (2)	4 (2)*	2 (2)*	4 (5)*	1 (2)*	-1 (2)*	0 (2)*	-2 (2)*

E373A RIG-I peptide sequence	charge	start	end	structure	(xv)	(xvi)	(xvii)	(xviii)	(xix)	(xx)	(xxi)	(xxii)
LESVSRDPSNENPKLED	3	597	613	HEL2	53 (2)	2 (4)*	1 (4)*	-2 (4)*	0 (4)*	-4 (5)*	-1 (4)*	-8 (4)
ESVSRDPSNENPKLED	3	598	613		52 (2)	1 (4)*	1 (4)*	-3 (3)*	0 (4)*	-4 (5)*	-1 (3)*	-8 (4)
VSRDPSNENPKLED	3	600	613		54 (3)	2 (5)*	1 (4)*	-2 (4)*	0 (5)*	-4 (5)*	-1 (4)*	-7 (4)
FILQEEYHLNPET	2	616	628		31 (2)	0 (2)*	0 (3)*	3 (2)*	-1 (2)*	-5 (2)*	-2 (3)*	-6 (2)
ILQEEYHLNPET	2	617	628		32 (2)	1 (2)*	2 (3)*	0 (2)*	0 (2)*	-3 (2)*	-1 (3)*	-6 (3)
ILQEEYHLNPETIT	2	617	630		33 (1)	0 (1)*	-1 (2)*	0 (1)*	-2 (1)*	-3 (2)*	-3 (2)*	-7 (2)
ILQEEYHLNPETITIL	2	617	632		24 (1)	0 (2)*	0 (3)*	-1 (2)*	0 (2)*	-3 (2)*	-1 (3)*	-5 (2)
EYHLNPET	2	621	628		53 (2)	0 (4)*	1 (3)*	0 (3)*	2 (3)*	-1 (3)*	1 (2)*	-4 (3)*
YHLNPET	2	622	628		61 (2)	0 (3)*	2 (3)*	-1 (3)*	-1 (3)*	-10 (4)	-2 (3)*	-15 (3)
FVKTRALVDALKNWIEGNPKL	3	633	653	motif IV	32 (1)	-1 (2)*	-1 (2)*	-5 (2)	-7 (2)	-19 (3)	-11 (4)	-19 (2)
FVKTRALVDALKNWIEGNPKL	4	633	653		32 (2)	-1 (2)*	-1 (2)*	-5 (2)	-5 (2)	-17 (3)	-10 (3)	-19 (2)
RALVDALKNWIEGNPKLSFLK	3	637	657		32 (1)	-1 (2)*	-1 (2)*	-3 (7)*	-7 (2)	-19 (3)	-11 (4)	-19 (2)
SFLKPGIL	2	654	661		41 (3)	4 (4)*	2 (5)*	3 (5)*	-2 (4)*	-7 (4)	-4 (5)*	-15 (5)
LKPGIL	2	656	661		64 (2)	4 (4)*	2 (7)*	0 (3)*	-4 (6)*	-11 (4)	-7 (7)	-25 (7)
TGRGKTNQNTGMTLPAQ	3	662	678		78 (4)	3 (7)*	1 (5)*	-1 (5)*	-3 (7)*	-2 (7)*	0 (6)*	0 (7)*
TGRGKTNQNTGMTLPAQK	4	662	679		78 (3)	1 (6)*	0 (6)*	-5 (4)	0 (4)*	-2 (6)*	0 (6)*	-2 (5)*
MTLPAQK	2	673	679		85 (3)	4 (4)*	0 (6)*	-4 (7)*	-3 (5)*	-1 (7)*	0 (6)*	-2 (7)*
DAFKASGDHNIL	2	683	694		47 (2)	2 (4)*	2 (3)*	1 (3)*	2 (4)*	-1 (4)*	1 (3)*	-1 (4)*
LIATSVADEGIDIAQCN	2	694	710	Pincer	75 (2)	5 (4)*	2 (3)*	3 (4)*	-1 (6)*	-15 (5)	-5 (3)	-39 (3)
IAQCNL	1	706	711		71 (2)	3 (4)*	1 (4)*	-2 (3)*	-1 (6)*	-11 (5)	-5 (4)*	-30 (3)
YEYVGNVIKM	2	715	724		86 (3)	4 (5)*	3 (4)*	3 (4)*	-1 (7)*	-12 (5)	-7 (4)	-34 (4)
LTSNAGVIEKEQINM	2	741	755		54 (2)	11 (4)	7 (3)	3 (3)*	-1 (5)*	-10 (6)	-10 (4)	-23 (3)
KEQINM	2	750	755		48 (2)	15 (4)	9 (3)	-2 (3)*	1 (4)*	-12 (4)	-8 (3)	-34 (3)
YKEKMMNDSIL	2	756	766		17 (3)	20 (4)	13 (5)	5 (4)*	0 (2)*	-5 (3)	-1 (4)*	-8 (4)
YKEKMMNDSIL	3	756	766		17 (4)	20 (4)	13 (5)	4 (4)*	1 (2)*	-6 (3)	0 (4)*	-8 (4)
SILRL	2	764	768		11 (4)	1 (5)*	0 (5)*	1 (4)*	-1 (3)*	-2 (3)*	3 (6)*	0 (5)*
SILRLQT	2	764	770		25 (3)	4 (3)*	2 (4)*	0 (4)*	2 (2)*	3 (2)*	7 (4)	8 (3)
RLQTWDEAV	2	767	775		53 (2)	3 (3)*	2 (3)*	1 (3)*	0 (4)*	-2 (6)*	5 (3)	5 (4)
RLQTWDEAVF	2	767	776		45 (2)	1 (2)*	2 (2)*	-4 (2)*	4 (2)*	5 (4)	5 (2)	7 (3)
DEAVF	1	772	776		24 (2)	-4 (3)*	-2 (3)*	4 (3)*	2 (7)*	-1 (4)*	-2 (4)*	-11 (4)
RDSQEKPKPVPDK	2	791	803	CTD	78 (2)	12 (5)	10 (4)	5 (3)*	-9 (6)	-20 (5)	-14 (4)	-23 (4)
KALACYTADVRVIE	3	814	827		45 (2)	-1 (4)*	-3 (4)*		-5 (6)*		-8 (5)	0 (2)*
VRVIE	2	823	827		33 (2)	2 (3)*	1 (3)*	0 (3)*	-1 (3)*	-2 (3)*	1 (3)*	-2 (3)*
VRVIEE	2	823	828		62 (3)	1 (3)*	2 (4)*	-2 (4)*	-2 (2)*	-3 (3)*	-1 (4)*	-2 (3)*
VRVIEECHY	3	823	831		40 (2)	-2 (2)*	2 (3)*	-6 (2)	0 (2)*	-3 (2)*	-1 (4)*	-3 (3)*
CHYTVLGDA	2	829	837		31 (1)	1 (2)*	0 (2)*	-3 (2)*	0 (2)*	2 (4)*	2 (2)*	0 (2)*
TVLGDA	1	832	837		52 (2)	1 (4)*	0 (3)*	-2 (3)*	0 (5)*	-1 (5)*	2 (3)*	0 (4)*
TVLGDAFKECF	2	832	842		51 (2)	-3 (3)*	-2 (3)*	-5 (3)*	1 (3)*	-1 (4)*	2 (3)*	1 (3)*
FKECF	2	838	842	Capping loop	38 (2)	-9 (3)	-5 (4)	-10 (4)	0 (3)*	-2 (3)*	0 (5)*	-4 (4)*
VSRPHPKPKQFSSF	3	843	856		77 (3)	-11 (5)	-1 (5)*	-13 (4)	-22 (6)	-7 (6)	-16 (5)	-3 (4)*
VSRPHPKPKQFSSF	4	843	856		77 (3)	-11 (5)	-2 (5)*	-11 (5)	-22 (5)	-7 (6)	-17 (4)	-2 (3)*
KPKQFSSFEKRA	2	849	860		59 (2)	5 (3)	3 (3)*	2 (2)*	-6 (5)	-10 (4)	-5 (3)	-3 (5)*
FEIPVIKIES	2	882	891		16 (2)	-7 (2)	3 (4)*	1 (2)*	1 (2)*	0 (2)*	0 (4)*	-3 (3)*
FEIPVIKIESF	2	882	892		16 (2)	-7 (2)	-5 (3)*	-7 (2)	0 (1)*	0 (1)*	-1 (2)*	-3 (2)*
FVVED	1	892	896		17 (1)	-5 (2)	-3 (4)*	-9 (2)	0 (3)*	-1 (2)*	0 (4)*	-3 (4)*
VVEDIATGVQTL	2	893	904	RNA binding region	69 (2)	-10 (3)	-6 (3)	-16 (3)	-1 (3)*	-5 (3)	-3 (4)*	-12 (3)
YSKWKDFHF	3	905	913		66 (2)	-11 (3)	-9 (5)	-10 (2)	-23 (3)	-32 (5)	-17 (6)	-35 (7)
YSKWKDFHFEKIPFDPAE	4	905	922		55 (2)	-9 (3)	-10 (3)	-11 (3)	-24 (5)	-35 (4)	-25 (4)	-33 (9)
EKIPFDPAE	2	914	922		43 (2)	-1 (3)*	0 (4)*	-5 (4)	-1 (3)*	-2 (4)*	1 (4)*	-1 (4)*
EKIPFDPAEMSK	2	914	925		59 (3)	-1 (5)*	0 (4)*	-4 (5)*	1 (5)*	-1 (6)*	1 (4)*	-2 (5)*
EKIPFDPAEMSK	3	914	925		60 (3)	-1 (5)*	0 (4)*	-3 (4)*	1 (5)*	-1 (6)*	2 (4)*	-1 (5)*

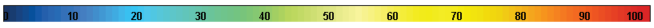

HDX Dynamics Key

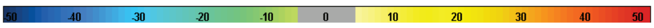

HDX Perturbation Key

C268F RIG-I peptide sequence	charge	start	end	structure	(xxiii)	(xxiv)	(xxv)	(xxvi)	(xxvii)	(xxviii)	(xxix)	(xxx)
FQDYIRKTLDPTYILS	3	12	27		38 (2)	23 (3)	17 (3)	8 (2)	1 (1)*	-5 (3)*	1 (2)*	1 (3)*
IRKTLDPTYIL	3	16	26		36 (2)	27 (3)	21 (3)	8 (3)	0 (2)*	-6 (3)		0 (3)*
SYMAPWFRE	2	27	35		47 (2)	21 (3)	20 (3)	8 (2)	-4 (3)*	-1 (3)*	-2 (4)*	-5 (4)*
YMAPWFREEEVQ	2	28	39		45 (2)	24 (3)	20 (3)	10 (2)	-3 (3)*	-4 (4)*	0 (3)*	-1 (3)*
VQYIQA	2	38	43		34 (2)		12 (3)	0 (4)*	-5 (2)*	0 (4)*	0 (2)*	-1 (2)*
YIQAEKNNKGPMEA	3	40	53	CARD1	47 (3)	12 (4)	10 (4)	-3 (3)*	-4 (4)*	-2 (4)*	-2 (6)*	-4 (4)*
LQEEGWFRGFL	2	64	74		52 (1)	19 (6)	7 (3)	0 (2)*	2 (5)*	-3 (5)*	1 (3)*	2 (3)*
FRGFLDA	2	70	76		15 (1)	23 (2)	21 (1)	5 (3)*	-1 (2)*	0 (2)*	0 (2)*	-2 (1)*
DALDHAGYSGL	2	75	85		28 (2)	19 (3)	15 (3)	5 (2)	-3 (3)*	-3 (3)*	0 (4)*	-1 (3)*
LDHAGYSGL	2	77	85		33 (3)	14 (4)	10 (4)	-2 (4)*	-3 (4)*	-4 (3)*	0 (5)*	-1 (3)*
YEAIESW	1	86	92		25 (2)	33 (3)	30 (3)	14 (4)	-1 (3)*	-2 (3)*	0 (2)*	-1 (2)*
YEAIESWD	1	86	93		26 (3)	30 (3)	26 (3)	12 (3)	-2 (3)*	-4 (3)*	-1 (2)*	-1 (3)*
FKKIEKLEE	3	94	102		64 (1)	9 (4)	6 (4)	2 (2)*	-3 (3)*	-1 (4)*	0 (0)*	-2 (4)*
YRLLL	2	103	107		9 (1)	28 (2)	25 (3)	8 (4)	-2 (2)*	-1 (2)*	-2 (1)*	-3 (4)*
YRLLLKRLQPEF	3	103	114	Latch region	12 (4)	41 (4)	37 (5)	23 (5)	-3 (3)*	-6 (2)	-1 (4)*	-3 (4)*
LLKRLQPEF	2	106	114		12 (4)	35 (5)	36 (4)	23 (4)	-2 (2)*	-4 (4)*	0 (3)*	-1 (3)*
LLKRLQPEF	3	106	114		16 (4)	46 (5)	40 (5)	22 (5)	-3 (3)*	-6 (4)	0 (4)*	-1 (4)*
KRLQPEF	2	108	114		21 (5)	38 (6)	42 (6)	23 (5)	-1 (1)*	-4 (4)*	-4 (4)*	-9 (5)
KRLQPEF	3	108	114		20 (5)	41 (6)	41 (6)	13 (6)	0 (1)*	-6 (3)	-2 (2)*	-6 (5)
KTRIIPTDIISDL	2	115	127	CARD2	51 (2)	22 (3)	17 (3)	8 (2)	2 (4)*	-6 (4)	-2 (2)*	1 (4)*
KTRIIPTDIISDL	3	115	127		51 (2)	22 (3)	17 (4)	7 (2)	-1 (4)*	-6 (4)	-5 (6)	1 (4)*
KTRIIPTDIISDLSE	3	115	129		58 (1)	21 (3)	13 (6)	4 (2)*	2 (4)*	-2 (4)*	2 (2)*	3 (4)*
ILQICSTKGMMAGA	2	139	152		37 (2)	30 (3)	25 (4)	12 (2)	-2 (4)*	-3 (3)*	2 (3)*	0 (4)*
ILQICSTKGMMAGAEKL	3	139	155		34 (4)	26 (4)	21 (4)	10 (4)	-2 (2)*	-2 (2)*	1 (1)*	0 (3)*
ILQICSTKGMMAGAEKLVE	3	139	157		26 (2)	29 (3)	26 (3)	13 (3)	-2 (2)*	-2 (3)*	1 (3)*	-1 (3)*
LLRSDKENWPKTLKL	4	159	173		28 (2)	32 (3)	26 (4)	14 (2)	-5 (4)*	-2 (4)*	-2 (3)*	0 (3)*
RSDKENWPKTLKLAL	4	161	175		26 (3)	24 (4)	14 (3)	18 (3)	2 (2)*	-3 (5)*	-1 (4)*	-3 (3)*
ALEKERNKFSEL	3	174	185		51 (2)	23 (4)	12 (3)	7 (3)	0 (1)*	-9 (4)	2 (2)*	1 (2)*
EKERNKFSEL	3	176	185		60 (2)	17 (3)	11 (4)	6 (2)	0 (3)*	-8 (4)	0 (1)*	0 (5)*
WIVEKGIKDVE	3	186	196		89 (3)	5 (5)*	-1 (6)*	-7 (5)	0 (5)*	-11 (5)	2 (6)*	3 (6)*
WIVEKGIKDVETEDL	3	186	200		92 (3)	3 (4)*	0 (5)*	-5 (5)	-1 (4)*	-8 (4)	-1 (6)*	2 (5)*
WIVEKGIKDVETEDLED	2	186	202		86 (3)	4 (4)*	0 (5)*	-6 (4)	5 (2)*	-11 (5)	1 (3)*	1 (6)*
WIVEKGIKDVETEDLEDKMETSD	3	186	208	linker	87 (2)	3 (3)*	0 (5)*	-7 (3)	3 (4)*	-8 (6)	1 (5)*	1 (4)*
IVEKGIKDVETEDL	3	187	200		85 (3)	4 (4)*	0 (5)*	-5 (4)*	-3 (6)*	-5 (4)	2 (5)*	1 (5)*
EDKMETSDIQIF	2	201	212		79 (2)	4 (4)*	1 (4)*	-3 (2)*	-2 (5)*	-3 (2)*	1 (5)*	2 (5)*
IQIFYQEDPECQNLSE	2	209	224		87 (3)	2 (6)*	-3 (6)*	-11 (4)	-2 (4)*	-14 (6)	2 (6)*	3 (6)*
FYQEDPECQNL	2	212	222		72 (3)	4 (5)*	0 (6)*	-14 (5)	-2 (6)*	-18 (6)	1 (6)*	2 (6)*
YQEDPECQNL	2	213	222		79 (4)	3 (5)*	0 (6)*	-17 (5)	-1 (6)*	-17 (6)	1 (7)*	1 (7)*
SENSCPPSEVSDTNL	2	223	237		78 (3)	3 (5)*	0 (6)*	-16 (5)	0 (5)*	-17 (5)	1 (5)*	2 (6)*
NSCPPSEVSDTNL	2	225	237		81 (4)	2 (4)*	-1 (5)*	-16 (5)	-2 (6)*	-15 (5)	1 (5)*	1 (6)*
VSDTNL	1	232	237		83 (3)	1 (5)*	0 (6)*	-13 (5)	0 (5)*	-9 (5)	1 (6)*	1 (6)*
YSPFKPRN	3	238	245		94 (3)	-3 (5)*	0 (7)*	-12 (5)	-4 (6)*	-6 (4)	-2 (6)*	
YSPFKPRNYQLE	3	238	249	HEL1	65 (3)	0 (3)*	-3 (4)*	-5 (3)*	-3 (4)*	-8 (4)	1 (4)*	6 (4)
YSPFKPRNYQLE	2	238	249		65 (2)	0 (3)*	-6 (4)	-5 (3)	-4 (4)*	-8 (3)	-2 (0)*	8 (5)
LICEHHLKKFPQGQKGKVVF	3	276	295		31 (3)	-14 (4)	1 (4)*	-3 (3)*	4 (1)*	-4 (3)*	-1 (2)*	-15 (5)
ICEHHLKKFPQGQKGKVVF	3	277	295		28 (1)	-13 (4)	3 (2)*	-2 (2)*	3 (3)*		-4 (3)*	-16 (3)
FANQIPVYEQQ	2	296	306		74 (3)	-42 (5)	-33 (4)	-29 (4)	-4 (4)*	-7 (4)	-1 (4)*	-18 (4)
FANQIPVYEQQKSVF	2	296	310	motif Ia	68 (3)	-37 (4)	-31 (4)	-21 (4)	-4 (4)*	-5 (4)*	0 (4)*	-15 (4)
FANQIPVYEQQKSVFSKY	3	296	313		62 (2)	-31 (3)	-27 (3)	-19 (3)	-3 (3)*		0 (3)*	-11 (3)
FERHGYRVTGISGATAENVPVEQIVENND	4	314	342		59 (1)	-1 (1)*	-5 (2)*	-9 (2)		-10 (4)	0 (4)*	
GYRVTGISGATAENVPVEQIVENN	4	318	341		60 (1)	0 (2)*	-2 (5)*	-9 (2)	-3 (2)*	-9 (3)	1 (4)*	-2 (2)*
YRVTGISGATAENVPVEQIVE	3	319	339		78 (3)	2 (4)*	-5 (2)	-9 (5)	-5 (4)	-10 (5)	1 (6)*	0 (6)*
IIILTPQIL	2	343	351		32 (1)	-15 (2)	-12 (2)	-15 (1)	-2 (2)*	-5 (1)*	-1 (2)*	-5 (2)
IIILTPQIL	1	343	351		32 (1)	-16 (2)	-13 (2)	-7 (1)	-2 (2)*	0 (2)*	-1 (2)*	-6 (2)
IIILTPQILVN	2	343	353		34 (1)	-16 (2)	-14 (2)	-22 (2)	-2 (2)*	-4 (2)*	0 (2)*	-5 (2)*
IIILTPQILVNNL	2	343	355		48 (2)	-26 (2)	-21 (3)	-13 (2)	-3 (3)*	-5 (3)	-2 (3)*	-10 (3)
IIILTPQILVNNLKKGTIPSL	3	343	363		64 (3)	-17 (3)	-16 (3)	-15 (3)	-4 (3)*	-11 (3)	-1 (3)*	-8 (3)
IIILTPQILVNNLKKGTIPSL	2	343	363	motif Ic	62 (3)	-18 (3)	-17 (4)	-15 (3)	-4 (3)*	-11 (3)	-1 (3)*	-8 (3)
IIILTPQILVNNLKKGTIPSLSI	3	343	365		61 (2)	-16 (3)	-14 (3)	-14 (3)	-4 (3)*	-12 (3)	-1 (3)*	-8 (3)
IIILTPQILVNNLKKGTIPSLSIF	3	343	366		59 (2)	-15 (3)	-14 (3)	-13 (3)	-3 (3)*	-17 (3)	-1 (3)*	-7 (3)
LMIFDECHNTSKQHPYNM	3	368	385		30 (2)	-11 (2)	-11 (3)	-5 (2)	-1 (4)*	-3 (3)*	1 (3)*	-1 (2)*
IFDECHNTSKQHPYNMIM	3	370	387	motif IIa	33 (2)	-11 (2)	-11 (3)	-6 (2)	-1 (3)*	-3 (3)*	0 (2)*	-1 (2)*
FNYLDQKLGGSSGPLPQVIG	2	388	407		36 (2)	1 (3)*	-1 (4)*	-2 (3)*	-2 (3)*	-5 (2)*	1 (3)*	3 (3)*
FNYLDQKLGGSSGPLPQVIGL	2	388	408		33 (3)	0 (3)*	-1 (4)*	-2 (3)*	-2 (3)*	-3 (3)*	1 (3)*	3 (3)*
DQKLGGSSGPLPQVIGL	2	392	408		37 (3)	1 (3)*	-2 (4)*	-3 (3)*	-2 (3)*	-3 (2)*	1 (3)*	3 (3)*
LTASVGVGDAKNTDEAL	2	408	424		63 (3)	-17 (3)	-17 (3)	-6 (8)	-2 (5)*	-3 (3)*	1 (9)*	-3 (3)*
TASVGVGDAKNTDEAL	2	409	424		64 (3)	-13 (4)	-14 (4)	-12 (4)	-3 (3)*	-7 (3)	0 (4)*	-3 (4)*
TASVGVGDAKNTDEALD	2	409	425		61 (3)	-14 (4)	-14 (5)	-10 (4)	-4 (3)*	-8 (3)	0 (4)*	-2 (4)*
DYICKL	2	425	430		-23 (2)	0 (2)*	0 (3)*	-1 (2)*	0 (2)*	1 (1)*	1 (3)*	0 (3)*
DYICKL	1	425	430		-11 (2)	-1 (2)*	0 (4)*	-1 (2)*	0 (3)*	2 (1)*	0 (4)*	0 (4)*
CASLDASVIATVKHNLEEL	2	431	449		35 (1)	-1 (1)*	-2 (2)*	-1 (1)*	-1 (2)*	-4 (1)*	1 (2)*	12 (2)
SVIATVKHNLEE	2	437	448		60 (2)	-5 (2)*	-6 (3)	-7 (4)	-1 (3)*	-5 (3)*	-2 (12)*	10 (4)
EQVVYKPQKF	3	450	459		69 (2)	-19 (3)	-16 (4)	-16 (3)	-3 (3)*	-1 (3)*	0 (3)*	21 (4)
VVYKPQKF	2	452	459		75 (3)	-14 (3)	-12 (4)	-12 (4)	-4 (3)*	-3 (4)*	1 (4)*	10 (4)
VVYKPQKF	3	452	459		75 (3)	-14 (4)	-10 (4)	-10 (4)	-4 (3)*	-3 (4)*	0 (4)*	8 (5)
FRKVESRISDKFKYIIAQL	3	460	478	HEL2i	32 (2)	-16 (3)	3 (3)*	-4 (2)*	0 (2)*	-4 (1)*	0 (2)*	1 (2)*
QLMRDTESL	2	477	485		47 (2)	-1 (4)*	0 (4)*	-14 (3)	-1 (3)*	-2 (1)*		-5 (3)*
MRDTESL	2	479	485		8 (1)	0 (1)*	0 (2)*	-2 (1)*	-1 (2)*	0 (1)*	0 (2)*	-1 (2)*
AKRICKDLENL	3	486	496		68 (1)	-2 (2)*	-6 (3)	-4 (3)*	3 (1)*	-7 (4)	1 (4)*	2 (3)*
SQIQNREFGTQ	2	497	507		79 (3)	-5 (6)	-7 (5)	-17 (4)	-2 (7)*	-2 (4)*	2 (5)*	0 (5)*
SQIQNREFGTQKYEQ	3	497	511		73 (2)	-6 (3)	-8 (3)	-9 (3)	-3 (4)*	-5 (4)*	1 (4)*	3 (4)*
SQIQNREFGTQKYEQW	3	497	512		65 (2)	-2 (5)*	-8 (4)	-4 (4)*	-2 (4)*	-2 (4)*	2 (4)*	5 (5)
WIVTVQKACM	2	512	521		9 (1)	-5 (2)	-8 (4)	0 (2)*	1 (3)*	1 (7)*	0 (4)*	1 (2)*
IVTVQKACM	2	513	521		12 (2)	-6 (2)	-7 (2)	-1 (2)*	-1 (2)*	1 (2)*	1 (2)*	0 (2)*
VFQMPDKDEESRICKA	3	522	537		58 (2)	-23 (5)	-18 (5)	-11 (2)	-3 (4)*	-7 (2)	-1 (3)*	-7 (3)
VFQMPDKDEESRICKALF	4	522	539	CTD:HEL2i	53 (2)	-18 (2)	-17 (3)	-9 (2)	0 (3)*	-7 (2)	-1 (3)*	-5 (3)
FQMPDKDEESRICKA	3	523	537		60 (2)	-19 (3)	-19 (4)	-11 (3)	-5 (3)*	-7 (4)	-3 (3)*	-5 (3)
LYTSHLRKYNDAL	4	540	552		9 (3)	1 (4)*	0 (4)*	2 (4)*	-3 (2)*	-2 (4)*	0 (3)*	0 (3)*
LYTSHLRKYNDAL	3	540	552		9 (3)	2 (4)*	0 (4)*	2 (4)*	-2 (3)*	-2 (4)*	0 (3)*	1 (4)*
IISEHARMKDALD	3	553	565		23 (1)	2 (3)*	-1 (2)*	-1 (2)*	-3 (6)*	-4 (3)*	0 (1)*	1 (2)*
HARMKDALD	2	557	565		34 (1)	1 (2)*	-4 (2)*	-3 (2)*	-2 (2)*	-4 (2)*	-3 (1)*	0 (2)*
YLKDFFSNV	2	566	574		16 (1)	14 (2)	12 (2)	6 (1)	-2 (2)*	-5 (2)	0 (2)*	0 (2)*
YLKDFFSNVRAAGF	3	566	579	Gate motif	40 (3)	20 (4)	12 (5)	2 (5)*	-3 (2)*	-15 (5)	-3 (14)*	2 (4)*
FFSNV	1	570	574		33 (2)	28 (2)	27 (3)	13 (2)	-4 (3)*	-5 (3)*	0 (4)*	-2 (4)*
FFSNVRAAGF	2	570	579		51 (2)	21 (4)	18 (5)	5 (3)*	-2 (5)*	-9 (5)	1 (5)*	-1 (5)*
DEIEQDLTQRFEEKLQE	3	580	596		17 (1)	10 (1)	6 (1)	3 (2)*	-2 (2)*	-7 (2)	-1 (3)*	-5 (3)
DEIEQDLTQRFEEKLQEL	3	580	597		22 (1)	9 (2)	6 (2)	3 (1)*	-2 (2)*	-10 (2)	0 (2)*	-3 (2)*
DEIEQDLTQRFEEKLQELES	3	580	599		24 (1)	8 (2)	5 (2)	1 (1)*	-2 (2)*	-10 (2)	0 (2)*	-4 (2)*
ESVSRDPSNENPKLEDLC	3	598	615		44 (2)	4 (2)*	4 (2)*	-4 (3)*	-1 (1)*	-10 (3)	2 (4)*	-2 (2)*
VSRDPSNENPKL	3	600	611		63 (4)	3 (5)*	1 (6)*	-8 (5)	-3 (5)*	-9 (4)	-2 (6)*	-9 (6)
VSRDPSNENPKLED	2	600	613		56 (3)	3 (4)*	0 (5)*	-8 (4)	-3 (4)*	-9 (3)	-1 (5)*	-5 (5)
FILQEEYHLNPET	2	616	628		26 (1)	0 (2)*	-2 (1)*	4 (2)*	-1 (2)*	-5 (2)	1 (2)*	-2 (2)*
ILQEEYHLNPETITIL	2	617	632	HEL2	22 (1)	0 (2)*	-3 (1)*	-2 (1)*	-1 (2)*	-4 (2)*	1 (1)*	-2 (2)*
EEYHLNPETITILFVKTR	2	620	637		37 (3)	0 (3)*	-2 (4)*	-2 (3)*	-2 (3)*	-4 (3)*	1 (3)*	3 (3)*
FVKTRALVDALKNWIEGNPKL	3	633	653		25 (1)	-12 (2)	-6 (1)	-5 (1)*	-1 (2)*	-6 (2)	-1 (2)*	-6 (1)
FVKTRALVDALKNWIEGNPKLSF	3	633	655	motif IV	24 (1)	-15 (2)	-4 (2)*	-4 (1)*	0 (2)*	-6 (4)	2 (3)*	
FVKTRALVDALKNWIEGNPKLSFLKPGIL	4	633	661		30 (1)	-18 (2)	-3 (2)*	-3 (2)*	-2 (2)*	-11 (3)	-5 (2)*	-12 (1)
SFLKPGIL	2	654	661		33 (4)	-2 (5)*	-5 (5)*	-5 (5)	-3 (3)*	-3 (5)*	-5 (5)	-13 (4)
KPGILTGRGKTNQNTG	3	657	672		50 (2)	-5 (3)*	-3 (3)*	-11 (3)	2 (3)*	-11 (3)	-2 (2)*	-9 (2)
TGRGKTNQNTGMTLPAQKCIL	4	662	682		78 (3)	2 (4)*	0 (6)*	-12 (5)	0 (3)*	-13 (5)	1 (4)*	0 (4)*
TGRGKTNQNTGMTLPAQKCIL	3	662	682		78 (3)	2 (4)*	-3 (1)*	-9 (5)	-1 (4)*	-10 (5)	1 (6)*	-1 (1)*
PAQKCIL	2	676	682		86 (2)	3 (5)*	2 (6)*	-10 (6)	0 (4)*	-7 (5)	-1 (6)*	2 (7)*
DAFKASGDHNIL	3	683	694		43 (3)	2 (2)*	-1 (3)*	-5 (3)*	-2 (4)*	-6 (3)	0 (4)*	1 (4)*
DAFKASGDHNIL	2	683	694		48 (2)	0 (4)*	-1 (4)*	0 (3)*	-1 (5)*	-6 (5)		-2 (5)*
FKASGDHNIL	3	685	694		29 (2)	3 (3)*	-1 (2)*	-4 (5)*	0 (3)*	-8 (3)	2 (3)*	3 (4)*

C268F RIG-I peptide sequence	charge	start	end	structure	(xxiii)	(xxiv)	(xxv)	(xxvi)	(xxvii)	(xxviii)	(xxix)	(xxx)
IATSVADEGID	2	695	705	Pincer	77 (2)	-6 (3)	-8 (5)	-20 (3)	-3 (4)*	-23 (3)	-3 (5)*	-38 (5)
IATSVADEGIDIAQCNL	2	695	711		78 (2)	-5 (3)*	-5 (4)*	-12 (3)	-4 (4)*	-26 (4)	-3 (4)*	-40 (4)
IATSVADEGIDIAQCNLVILYEYVGNVIKM	3	695	724		66 (2)	-8 (2)	-6 (3)	-12 (3)	-2 (3)*	-23 (2)	-2 (3)*	-45 (3)
VILYEYVGNVIKM	2	712	724		68 (2)	-21 (2)	-18 (2)	-13 (3)	-3 (3)*	-19 (4)	-2 (2)*	-11 (2)
YEYVGNVIKM	2	715	724		86 (2)	-27 (3)	-21 (3)	-12 (3)	-4 (3)*	-8 (3)	-3 (3)*	-17 (3)
YVGNVIKM	2	717	724		84 (2)	-28 (3)	-21 (3)	-12 (3)	-3 (3)*	-6 (3)	-4 (3)*	-17 (3)
IQTRGRGRARGSKC	4	725	738		77 (4)	-21 (4)	-15 (4)	-20 (5)	-5 (3)	-18 (4)	-2 (5)*	-20 (5)
FLLTSNAGVIE	2	739	749		57 (2)	-21 (2)	-14 (3)	-19 (2)	3 (2)*	0 (1)*	2 (2)*	
FLLTSNAGVIEKEQINM	2	739	755		58 (1)	-28 (2)	-26 (1)	-24 (4)	-2 (2)*	-4 (3)*	0 (2)*	0 (2)*
LTSNAGVIEKEQINM	2	741	755		57 (1)	-31 (3)	-28 (2)	-22 (2)	-1 (3)*	-3 (2)*	-1 (3)*	0 (3)*
KEQINM	2	750	755		58 (2)	-38 (4)	-33 (5)	-23 (3)	-2 (4)*	-3 (3)*	-1 (4)*	-2 (5)*
YKEKMMNDSIL	3	756	766		21 (3)	-4 (4)*	-6 (4)	0 (4)*	0 (1)*	0 (4)*	0 (4)*	5 (3)
YKEKMMNDSIL	2	756	766		21 (3)	-4 (4)*	-6 (4)	-2 (4)*	1 (3)*	-1 (4)*	0 (4)*	5 (4)*
SILRLQT	2	764	770		27 (3)	-4 (3)*	-3 (5)*	-4 (3)*	-1 (3)*	-3 (3)*	1 (4)*	10 (2)
RLQTWDEAVF	2	767	776		46 (1)	-6 (2)	-7 (2)	-4 (2)*	1 (2)*	2 (2)*	2 (3)*	7 (2)
WDEAVF	1	771	776		66 (3)	-1 (3)*	-2 (3)*	-6 (4)	-2 (4)*	-4 (2)*	2 (3)*	3 (3)*
RDSQEKPKPVPDK	2	791	803	CTD	90 (2)	-13 (3)	-18 (5)	-12 (2)	-3 (3)*	-7 (3)	1 (3)*	1 (2)*
VRVIEE	2	823	828		59 (1)	1 (2)*	3 (2)*	-12 (2)	-1 (2)*	-7 (2)	-1 (2)*	-2 (2)*
VRVIEECHY	3	823	831		43 (1)	-1 (2)*	0 (3)*	-8 (1)	-2 (3)*	-10 (3)	1 (3)*	0 (3)*
VRVIEECHYTVLGDA	3	823	837		34 (1)	-1 (2)*	2 (0)*	-11 (1)	-1 (2)*	-9 (2)	-1 (2)*	-1 (2)*
CHYTVLGDA	2	829	837		30 (1)	1 (2)*	2 (2)*	-3 (2)*	-1 (2)*	-3 (1)*	0 (2)*	-2 (2)*
TVLGDAFKECF	2	832	842		52 (2)	-4 (3)*	-3 (3)*	-8 (3)	-3 (3)*	-14 (3)	0 (3)*	-1 (3)*
FKECF	1	838	842		42 (3)	-8 (4)	-7 (4)	-10 (3)	-3 (4)*	-9 (4)	-2 (5)*	-4 (4)*
FKECF	2	838	842		39 (3)	-8 (4)	-6 (4)	-12 (4)	-4 (4)*	-2 (4)*	-1 (4)*	-5 (4)*
FVSRPHPKPKQFSS	4	842	855	Capping loop	79 (3)	-6 (4)	-4 (5)*	-14 (4)	-4 (4)*	-10 (4)	0 (4)*	2 (5)*
VSRPHPKPKQFSSF	4	843	856		77 (3)	-6 (4)	-4 (5)*	-14 (4)	-4 (4)*	-10 (4)	2 (5)*	4 (6)*
VSRPHPKPKQFSSF	3	843	856		75 (2)	-7 (4)	-4 (5)*	-18 (4)	-2 (4)*	-11 (5)	2 (4)*	3 (6)*
EKRAKIF	2	857	863		49 (2)	-13 (3)	-13 (3)	-14 (3)	-3 (0)*	-4 (4)*	-1 (4)*	-3 (3)*
EKRAKIFC	3	857	864		26 (2)	-11 (3)	-12 (4)	-12 (3)	-2 (2)*	0 (5)*	-2 (0)*	-4 (4)*
EKRAKIFC	2	857	864		48 (3)	-10 (4)	-14 (3)	-19 (3)	-1 (2)*	-3 (2)*	0 (1)*	3 (4)*
CARQNCSHDWGIHVKYKT	4	864	881		35 (2)	-16 (3)	-2 (2)*	-8 (2)	-2 (3)*	2 (5)*	-3 (3)*	3 (4)*
FEIPVIKIES	2	882	891		14 (1)	-10 (2)	-6 (1)	-9 (1)	0 (1)*	1 (1)*	4 (6)*	1 (2)*
FEIPVIKIESF	2	882	892		18 (1)	-8 (2)	-8 (2)	-8 (1)	0 (1)*	1 (1)*	0 (1)*	0 (1)*
EIPVIKIESF	2	883	892		14 (1)	-6 (3)	-6 (1)	-9 (1)	0 (1)*	1 (1)*	4 (6)*	1 (2)*
FVVEDIATGVQTL	2	892	904	RNA binding region	63 (2)	-16 (3)	-17 (2)	-23 (2)	-3 (3)*	-21 (4)	0 (3)*	-2 (3)*
VVEDIATGVQTL	2	893	904		72 (2)	-19 (3)	-17 (2)	-24 (2)	-3 (3)*	-11 (3)	-2 (2)*	-5 (3)
VVEDIATGVQTL	1	893	904		71 (2)	-19 (3)	-20 (3)	-19 (2)	-3 (3)*	-9 (4)	-1 (3)*	-3 (3)*
EKIPFDPAE	2	914	922		51 (4)	-1 (4)*	-1 (5)*	-7 (4)	-3 (4)*	-9 (3)	0 (5)*	-3 (4)*
EKIPFDPAEMSK	2	914	925		61 (4)	-2 (4)*	-4 (6)*	-10 (4)	-3 (5)*	-9 (4)	0 (6)*	-1 (5)*
EKIPFDPAEMSK	3	914	925		62 (4)	-2 (4)*	-4 (6)*	-10 (4)	-3 (5)*	-6 (3)	0 (5)*	-1 (5)*

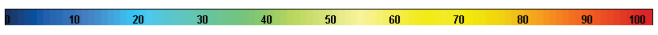

HDX Dynamics Key

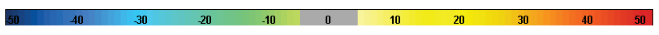

HDX Perturbation Key

Supplementary Figure 1

A summary of all HDX experiments. (**a**) Schematic representations of all HDX characterizations performed in this study. (**b**) HDX consolidation view for all studied conformational states of WT/H830A/E373A/C268F RIG-I with the experimental order in **Supplementary Fig 1a**. (**c**) HDX Experimental view for all studied conformational states of WT/H830A/E373A/C268F RIG-I with the experimental order in **Supplementary Fig 1a**. In Supplementary Fig 1c, specific RIG-I domain/motifs are defined in Fig 1a (CARD2 latch region is the region that intra-molecularly interacts with HEL2i gate motif in the apo receptor state. In HEL2i, the CTD:HEL2i region is the HEL2i region that CTD clashes with upon RNA agonist binding).

The values listed under each HDX experiments demonstrate the averaged difference in percentage of deuterium incorporation of that corresponding peptide either derived from apo protein or between two different states across all exchange time points (i.e., 0s, 10s, 60s, 300s, 600s, 900s, 1500s and 3600s). Including time 0s there were 8 time points and each time point was replicated with two independent MS runs per conformational state of receptor or receptor complex. HDX analyses were performed duplicate or triplicate, with single preparations of each protein ligand complex. The intensity weighted mean m/z centroid value of each peptide envelope was calculated and subsequently converted into a percentage of deuterium incorporation. Corrections for back-exchange were made on the basis of an estimated 70% deuterium recovery, and accounting for the known 80% deuterium content of the deuterium exchange buffer. When comparing the two samples, the perturbation %D is determined by calculating the difference between the two samples. HDX Workbench colors each peptide according to the smooth color gradient HDX perturbation key (D%) shown in each indicated figure. Differences in %D between -5% to 5% are considered non-significant and are colored gray according to the HDX perturbation key [1]. In addition to the -5% to 5% test, Unpaired t-tests are calculated to detect statistically significant ($p<0.05$) differences between samples at each time point. At least one time point with a p value less than 0.05 was present in the data set further confirming that the difference is significant. A negative value represents decreased deuterium incorporation or

stabilization while a positive value represents increased deuterium incorporation or de-stabilization in the corresponding region of the receptor when a binding event takes place. Peptides exhibiting statistically insignificant or undetectable changes are colored gray. Blank region represents undetected peptide for corresponding experiment. The deuterium incorporation data from all overlapping peptides were consolidated to individual amino acid values using a residue averaging approach [2]. HDX Workbench was used to automate mapping of gradient colors to pymol structure model [3]. For HDX experiments – i, ii, xii, xv and xxiii – measuring apo receptor dynamics, colors are coded according to HDX dynamics key. For the rest differential HDX experimental comparison views, colors are coded according to HDX perturbation key.

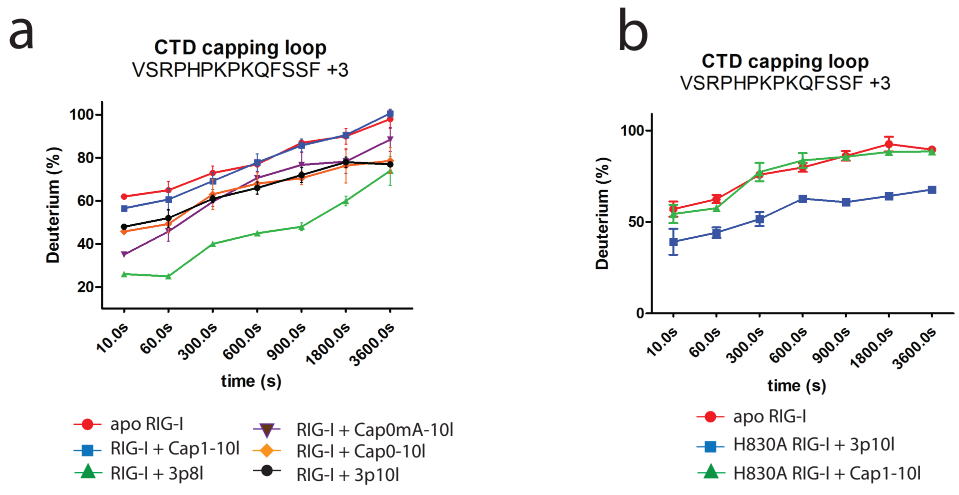

Supplementary Figure 2

C

sequence	charge	start	end	structure	RIG-I ± 3p8l
QDYIRKTLDPTYIL	3	13	26		1 (2)*
IRKTLDPTYIL	3	16	26		0 (3)*
SYMAPWFRE	2	27	35		3 (2)*
YMAPWFREEEVQ	2	28	39		3 (2)*
VQYIQA	2	38	43		1 (2)*
YIQAEKNNKGPMEA	3	40	53		-1 (4)*
EKNNKGPMEAATL	2	44	56		-1 (3)*
LQEEGWFRGFL	2	64	74		1 (3)*
QEEGWFRGFL	2	65	74		2 (3)*
FRGFLDA	2	70	76		3 (1)*
DALDHAGYSGL	2	75	85		1 (4)*
LDHAGYSGL	2	77	85		0 (6)*
YEAIESW	1	86	92		-1 (1)*
YEAIESWD	1	86	93		-1 (2)*
FKKIEKLEE	3	94	102		0 (3)*
YRLLL	2	103	107		11 (4)
YRLLLKRLQPEF	3	103	114	latch region	15 (4)
LLKRLQPEF	2	106	114		15 (5)
LLKRLQPEF	3	106	114		22 (6)
KRLQPEF	2	108	114		21 (6)
KRLQPEF	3	108	114		22 (5)
KTRIIPTDIISD	3	115	126		4 (3)*
KTRIIPTDIISDL	2	115	127		3 (3)*
KTRIIPTDIISDL	3	115	127		3 (3)*
KTRIIPTDIISDLSE	3	115	129		3 (2)*
EEILQICSTKGMMAGAEKL	3	137	155		8 (2)
ILQICSTKGMMAGA	2	139	152		7 (2)
ILQICSTKGMMAGAEKL	3	139	155	CARD2	5 (2)
ILQICSTKGMMAGAEKLVE	3	139	157		8 (2)
LLRSDKENWPKTLKL	4	159	173		1 (1)*
RSDKENWPKTLKLAL	4	161	175		5 (2)
ALEKERNKFSEL	3	174	185		3 (4)*
ALEKERNKFSEL	4	174	185		3 (3)*
EKERNKFSEL	3	176	185		2 (4)*
WIVEKGIKDVE	3	186	196		0 (5)*
WIVEKGIKDVETEDL	3	186	200		0 (6)*
WIVEKGIKDVETEDLEDKMETSD	3	186	208		-1 (5)*
IVEKGIKDVETEDL	3	187	200		0 (1)*
EDKMETSDIQIF	2	201	212		0 (4)*
FYQEDPECQNL	2	212	222		-1 (3)*
YQEDPECQNL	2	213	222		0 (4)*
SENSCPPSEVSDTNL	2	223	237		0 (6)*
NSCPPSEVSDTNL	2	225	237		-1 (6)*
VSDTNL	1	232	237		0 (5)*
YSPFKPRN	3	238	245		0 (5)*
YSPFKPRNYQLE	2	238	249		4 (5)*
YSPFKPRNYQLE	3	238	249		4 (5)*
LALPAMKGKNTIIC	2	250	263		1 (1)*
LALPAMKGKNTIICAPTGCGKTFVSLL	3	250	276		1 (4)*
LALPAMKGKNTIICAPTGCGKTFVSLL	4	250	276		-2 (2)*
APTGCGKTF	2	264	272		1 (4)*
PQGQKGKVVF	3	286	295		-1 (3)*
FANQIPVYEQQ	2	296	306		0 (4)*
FANQIPVYEQQKSVF	2	296	310		-3 (5)*
FANQIPVYEQQKSVFSKY	3	296	313		0 (2)*
FERHGYRVTGISGATAENVPVEQIVENND	4	314	342		-1 (2)*
GYRVTGISGATAENVPVEQIVENN	4	318	341		0 (3)*
YRVTGISGATAENVPVEQIVE	3	319	339		1 (4)*
IIILTPQIL	1	343	351		-3 (1)*
IIILTPQIL	2	343	351		-3 (1)*
IIILTPQILVN	2	343	353		-6 (1)
IIILTPQILVNN	2	343	354	motif Ic	-6 (2)
IIILTPQILVNNL	2	343	355		-5 (2)*
IIILTPQILVNNLKKGTIPSL	2	343	363		-3 (5)*
IIILTPQILVNNLKKGTIPSL	3	343	363		-3 (5)*
IIILTPQILVNNLKKGTIPSLSI	3	343	365		-3 (5)*
IIILTPQILVNNLKKGTIPSLSIF	3	343	366		-3 (4)*
KGTIPSLSIFT	2	357	367		0 (2)*
KGTIPSLSIFT	3	357	367		0 (2)*
LMIFDECHNTSKQHPYNM	3	368	385		-2 (2)*
IFDECHNTSKQHPYNMIM	2	370	387		-3 (3)*
IFDECHNTSKQHPYNMIM	3	370	387		-2 (2)*
FNYLDQKLGGSSGPLPQVIG	2	388	407		0 (1)*
FNYLDQKLGGSSGPLPQVIGL	2	388	408		0 (1)*
DQKLGGSSGPLPQVIGL	2	392	408		0 (2)*
LTASVGVGDAKNTDEAL	2	408	424		0 (3)*
TASVGVGDAKNTDEAL	2	409	424		1 (4)*
TASVGVGDAKNTDEALD	2	409	425		0 (5)*
TASVGVGDAKNTDEALDYICKL	3	409	430		3 (2)*
DYICKL	1	425	430		0 (1)*
DYICKL	2	425	430		0 (0)*
CASLDASVIATVKHNLEE	2	431	448		1 (4)*
CASLDASVIATVKHNLEEL	2	431	449		-1 (1)*
SVIATVKHNLEE	2	437	448		-1 (1)*
EQVVYKPQKF	3	450	459		0 (3)*
VVYKPQKF	2	452	459		2 (3)*
VVYKPQKF	3	452	459		2 (3)*
MRDTESL	2	479	485		0 (1)*
AKRICKDLENL	3	486	496		1 (3)*
SQIQNREFGTQ	2	497	507		1 (4)*
SQIQNREFGTQKYEQ	3	497	511		0 (6)*
SQIQNREFGTQKYEQW	3	497	512		0 (5)*
WIVTVQKACM	2	512	521		0 (5)*
IVTVQKACM	2	513	521		0 (1)*
VFQMPDKDEESRICKA	3	522	537		0 (5)*
VFQMPDKDEESRICKALF	4	522	539		0 (4)*
FQMPDKDEESRICKA	3	523	537		1 (4)*
FQMPDKDEESRICKALF	4	523	539		1 (4)*
LYTSHLRKYNDAL	3	540	552		0 (4)*
LYTSHLRKYNDAL	4	540	552		0 (4)*
YTSHLRKYNDAL	2	541	552		0 (4)*
IISEHARMKDALD	3	553	565		0 (1)*
HARMKDALD	2	557	565		-1 (2)*
HARMKDALD	3	557	565		0 (1)*
DYLKDFFSNVRAAGF	3	565	579		2 (3)*
YLKDFFSNV	2	566	574		0 (0)*
YLKDFFSNVRAAGF	3	566	579		-1 (3)*
FFSNV	1	570	574		1 (2)*
FFSNVRAAGF	2	570	579		1 (2)*
AAGFDEIEQDLT	2	576	587		-2 (3)*
DEIEQDLTQRFEEKLQE	3	580	596		2 (1)*
DEIEQDLTQRFEEKLQEL	3	580	597		2 (1)*
DEIEQDLTQRFEEKLQELES	3	580	599		2 (1)*
QELESVSRDPSNENPKLED	3	595	613		-1 (5)*
ESVSRDPSNENPKLEDLC	3	598	615		0 (6)*
VSRDPSNENPKL	3	600	611		2 (3)*
VSRDPSNENPKLED	2	600	613		0 (5)*
FILQEEYHLNPET	2	616	628		-1 (2)*
ILQEEYHLNPETITIL	2	617	632		0 (1)*
EEYHLNPETITILFVKTR	2	620	637		0 (2)*
FVKTRALVDALKNWIEGNPKL	3	633	653		0 (3)*
WIEGNPKL	2	646	653		0 (4)*
WIEGNPKLSFLKPGIL	2	646	661		1 (5)*
SFLKPGIL	2	654	661		0 (6)*
KPGILTGRGKTNQNTG	3	657	672		1 (3)*
TGRGKTNQNTGMTLPAQKCIL	3	662	682		1 (4)*
TGRGKTNQNTGMTLPAQKCIL	4	662	682		1 (4)*
PAQKCIL	2	676	682		1 (2)*
DAFKASGDHNIL	2	683	694		0 (5)*
DAFKASGDHNIL	3	683	694		0 (5)*

sequence	charge	start	end	structure	RIG-I ± 3p8l
FKASGDHNIL	3	685	694		0 (5)*
IATSVADEGID	2	695	705		0 (5)*
IATSVADEGIDIAQCNL	2	695	711		-5 (4)*
IATSVADEGIDIAQCNLVILYEYVGNVIKM	3	695	724		0 (6)*
SVADEGIDIAQ	2	698	708		-1 (4)*
VILYEYVGNVIKM	2	712	724		2 (4)*
YEYVGNVIKM	2	715	724		0 (4)*
YVGNVIKM	2	717	724		0 (4)*
IQTRGRGRARGSKC	4	725	738		1 (4)*
FLLTSNAGVIE	2	739	749		1 (3)*
FLLTSNAGVIEKEQINM	2	739	755		1 (4)*
LTSNAGVIEKEQINM	2	741	755		0 (5)*
AGVIEKE	2	745	751		-1 (5)*
KEQINM	2	750	755		0 (4)*
YKEKMMNDSIL	2	756	766		3 (2)*
YKEKMMNDSIL	3	756	766		2 (3)*
SILRLQT	2	764	770		3 (2)*
RLQTWDEAVF	2	767	776		-2 (2)*
WDEAVF	1	771	776		-3 (2)*
FREKILHIQTHEKF	4	776	789		1 (1)*
THEKFIRDS	2	785	793		0 (0)*
EKFIRDSQEK	2	787	796	CTD	2 (3)*
VRVIEE	2	823	828		-5 (2)*
VRVIEECHY	2	823	831	RNA binding region	-6 (2)
VRVIEECHY	3	823	831		-8 (1)
VRVIEECHYTVLGDA	3	823	837		-3 (1)*
CHYTVLGDA	2	829	837		-1 (2)*
TVLGDAFKECF	2	832	842	RNA binding region	-12 (5)
FKECF	1	838	842		-25 (5)
FKECF	2	838	842		-27 (5)
FVSRPHPKPKQFSS	4	842	855		1 (5)*
VSRPHPKPKQFSSF	3	843	856		2 (5)*
VSRPHPKPKQFSSF	4	843	856		1 (5)*
EKRAKIF	2	857	863	RNA binding region	-12 (4)
EKRAKIFC	3	857	864		-12 (2)
CARQNCSHDWGIHVKYKT	4	864	881		-3 (1)*
DWGIHVKYKTFEIPVIKIESFVV	4	872	894		-2 (3)*
FEIPVIKIES	2	882	891	RNA binding region	-19 (3)
FEIPVIKIESF	2	882	892		-32 (1)
EIPVIKIESF	2	883	892		-19 (3)
FVVEDIATGVQTL	2	892	904		-3 (2)*
VVEDIATGVQTL	1	893	904		-4 (4)*
VVEDIATGVQTL	2	893	904		-4 (3)*
IATGVQTL	2	897	904		3 (5)*
YSKWKDFHFEKIPFDPAE	3	905	922		2 (3)*
EKIPFDPAEMSK	2	914	925		-3 (4)*
EKIPFDPAEMSK	3	914	925		-4 (5)*

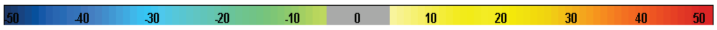

HDX perturbation key

Supplementary Figure 2

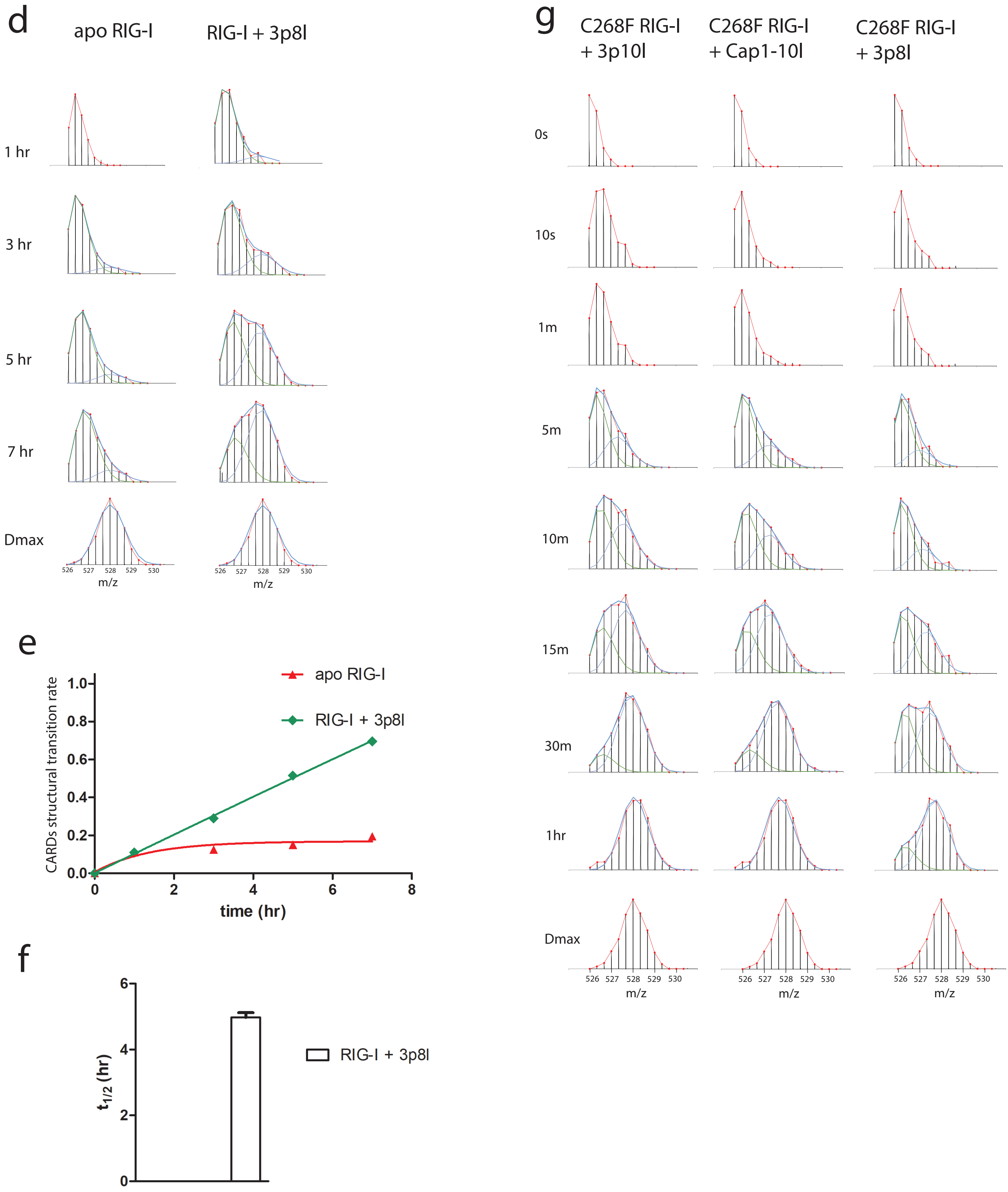

Supplementary Figure 2

Supplementary Figure 2

Differential deuterium uptake plots of CTD capping loop peptide region (VSRPHPKPKQFSSF, +3) of WT (a) and H830A RIG-I (b) upon receptor perturbed by indicated RNAs. The data are plotted as percent deuterium uptake verse time on a logarithmic scale. The HDX plots of this CTD capping loop peptide between indicated groups were statistically analysis by HDX Workbench (Supplementary Fig. 1c) [1]. (c) HDX perturbation view of RIG-I with and without 3p8l in longer HDX-MS time points (3 hr and 5 hr). This HDX perturbation view is presented as Supplementary Fig. 1c. (d) MS spectra of RIG-I CARD2 latch peptide Y103-114 derived from indicated complexes in indicated on-exchange time points. The quantity of each mass population was determined as Fig. 2a. (e) In each indicated state, the fraction of RIG-I CARDs molecules in the higher MS population (open conformation) to the total amount is plotted against the incubation HDX time points as Fig.2c. (f) Half-life ($t_{1/2}$) of respective partial unfolding event is determined by fitting an exponential curve as Fig. 2d. No half-life ($t_{1/2}$) of partial unfolding event is predicted I apo RIG-I in the indicated time points. (g) MS spectra of C268F RIG-I CARD2 latch peptide Y103-114 derived from indicated complexes in indicated on-exchange time points. The quantity of each mass population was determined as Fig. 2a.

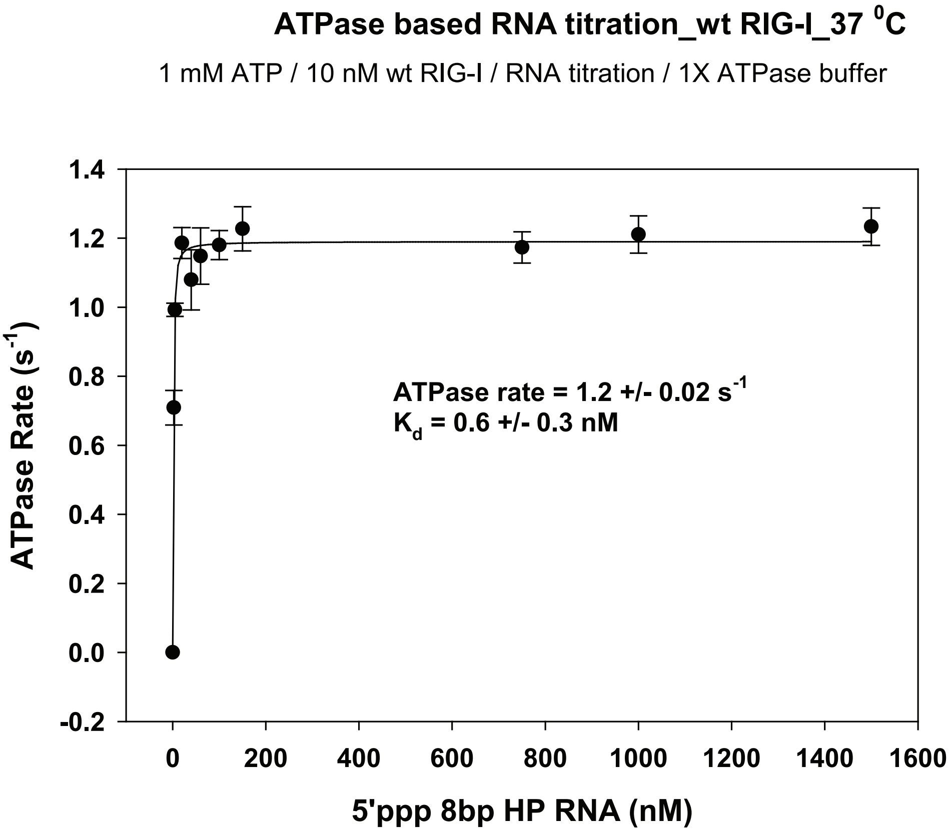

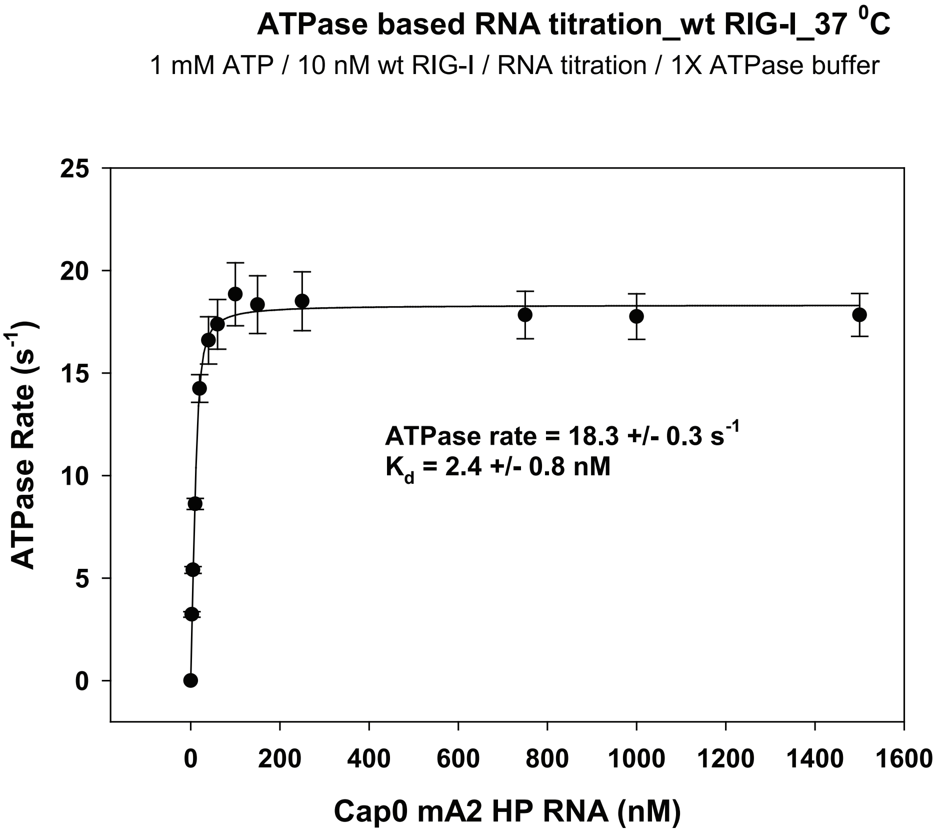

Supplementary Figure 3

Supplementary Figure 3 (related to Table 1)

K_d, ATPase hydrolysis activity of WT RIG-I for 3p8l and Cap0mA-10l. The data is derived from radiometric ATPase assay wherein a fixed concentration of protein is titrated with increasing concentration of RNA. The curves are fit to the quadratic equation to obtain the k_{atpase} and $k_{d,\ app}$ values. Error bars are from the time courses of the ATPase rate measurements at each RNA concentration.

Supplementary Table 1. Primers for generating RIG-I mutants

RIG-I mutants	Prime sequence
**H830A**	5'-GAGTGATAGAGGAATGCGCTTACACTGTGCTTGGAGATG-3'<br>5'-CATCTCCAAGCACAGTGTAAGCGCATTCCTCTATCACTC-3'
**E373A**	5'-CTTTGATGATATTTGATGCGTGCCACAACACTAGTAAACAAC-3'<br>5'-GTTGTTTACTAGTGTTGTGGCACGCATCAAATATCATCAAAG-3'
**C268F**	5'-ATATGTGCTCCTACAGGTTTTGGAAAAACCTTTGTTTCACTGC-3'<br>5'-GCAGTGAAACAAAGGTTTTTCCAAAACCTGTAGGAGCACATAT-3'

References:

1. Pascal, B.D. et al. HDX workbench: software for the analysis of H/D exchange MS data. *J Am Soc Mass Spectrom* **23**, 1512-21 (2012).
2. Keppel, T.R. & Weis, D.D. Mapping residual structure in intrinsically disordered proteins at residue resolution using millisecond hydrogen/deuterium exchange and residue averaging. *J Am Soc Mass Spectrom* **26**, 547-54 (2015).
3. Zheng, J. et al. High-resolution HDX-MS reveals distinct mechanisms of RNA recognition and activation by RIG-I and MDA5. *Nucleic Acids Res* **43**, 1216-30 (2015).
